# Using the online version of the Trier Social Stress Test to investigate the effect of acute stress on functional lateralization

**DOI:** 10.1038/s41598-024-71668-w

**Published:** 2024-09-06

**Authors:** Lena Sophie Pfeifer, Katrin Heyers, Oliver T. Wolf, Ursula Stockhorst, Onur Güntürkün, Christian J. Merz, Sebastian Ocklenburg

**Affiliations:** 1 Cognitive Psychology, Institute of Cognitive Neuroscience, Faculty of Psychology, Ruhr University Bochum, Bochum, Germany; 2 Biopsychology, Institute of Cognitive Neuroscience, Faculty of Psychology, Ruhr University Bochum, Bochum, Germany; 3 Experimental Psychology II and Biological Psychology, Institute of Psychology, School of Human Sciences, Osnabrück University, Osnabrück, Germany; 4 Department of Psychology, MSH Medical School Hamburg, Hamburg, Germany; 5 Institute for Cognitive and Affective Neuroscience, MSH Medical School Hamburg, Hamburg, Germany

**Keywords:** Stress, Online stressor, TSST-OL, Functional hemispheric asymmetries, Dichotic listening, Line bisection task, Psychology, Biomarkers

## Abstract

How stress affects functional hemispheric asymmetries is relevant because stress represents a risk factor for the development of mental disorders and various mental disorders are associated with atypical lateralization. Using three lateralization tasks, we investigated whether functional hemispheric asymmetries in the form of hemispheric dominance for language (verbal dichotic listening task), emotion processing (emotional dichotic listening task), and visuo-spatial attention (line bisection task) were affected by acute stress in healthy adults. One hundred twenty right-handed men and women performed these lateralization tasks in randomized order after exposure to a mild online stressor (i.e., an online variant of the Trier Social Stress Test (TSST), TSST-OL) and a non-stressful online control task (friendly TSST-OL, fTSST-OL) in a within-subjects design. Importantly, the verbal and the emotional dichotic listening tasks were presented online whereas the line bisection task was completed in paper–pencil form. During these tasks, we found the expected hemispheric asymmetries, indicating that online versions of both the verbal and the emotional dichotic listening task can be used to measure functional hemispheric asymmetries in language and emotion processing remotely. Even though subjective and physiological markers confirmed the success of the online stress manipulation, replicating previous studies, we found no stress-induced effect on functional hemispheric asymmetries. Thus, in healthy participants, functional hemispheric asymmetries do not seem to change flexibly in response to acute stress.

## Introduction

Lateralization is a fundamental characteristic of the brain’s structure and function^1^. Comparing the two halves of the brain, asymmetries can be found in the size and anatomical organization of gray^2^ and white matter structures^3^, as well as in various cognitive functions^4–6^. For instance, even though the classic model has undergone some revision^7^, the neural network involved in language processing is lateralized with two left-hemispheric structures, Broca’s area and Wernicke’s area, representing key structures^8,9^. Against this background, atypical lateralization manifests in increased bilateral activation of the brain that originates from an alteration in direction (reversal) or strength (reduction) of asymmetries. With that, altered asymmetries functionally imply increased activation of the hemisphere that is typically not dominant for a certain process while the dominant hemisphere shows decreased activation^10^. Importantly, atypical lateralization has been observed in various mental disorders.

One factor that has been associated with both mental disorders and atypical lateralization is stress^11^. Stress occurs when external demands exceed internal resources in a way that an individual is not able to handle a stressor at hand^12^. The Trier Social Stress Test (TSST,^13^) is a laboratory stress induction protocol that serves to investigate acute stress and the resulting stress response in experimental research. In a nutshell, the TSST represents a mock job interview in which participants apply for their dream job in front of a social-evaluative, non-responsive jury of researchers. The resulting acute stress response covers the activation of two physiological systems: the sympathetic nervous system (SNS) and the hypothalamic–pituitary–adrenal (HPA) axis^14,15^. The stress response is adaptive in releasing energy to make the individual adequately reactive in the face of an acute stressor^15^. However, chronic or repeated stress may result in certain dynamics of dysfunctional reactivity which are associated with poor health outcomes^16^. Thus, stress is considered an unspecific factor in the development and progression of mental and somatic diseases^17–19^.

Combining these two lines of evidence leads to the question of whether stress and atypical lateralization may be integrated into a triad alongside clinical outcomes^20,21^. In particular, Bishop^22^ proposed four theoretical scenarios on how atypical lateralization and clinical outcomes may be related to each other. These scenarios partly model a causal relation in which atypical lateralization and clinical outcomes may occur as causes or consequences of one another. Alternatively, atypical lateralization and clinical outcomes may occur as correlates. In this case, a common source may produce atypical lateralization as well as clinical outcomes. Bishop^22^ discussed genetic factors to initiate the proposed dynamics. However, also stress might function as a common source that triggers atypical lateralization and clinical outcomes. At least, in such a trajectory, stress might act as an environmental trigger in a gene-environment interaction model^20^. For instance, individuals who were exposed to pre- or perinatal stress show atypical lateralization with higher prevalence (e.g.,^23^). Moreover, in post-traumatic stress disorder (PTSD) which arises out of an extremely stressful experience by definition, studies reported not only reduced basal cortisol levels (for a meta-analysis, see^24^) but also altered functional lateralization (e.g., handedness,^25^). In this context, stress might contribute to altered structural and/or functional hemispheric asymmetries which in turn mediate the development of psychopathology in a (a) non-specific, (b) diagnosis-specific, or (c) symptom-specific way^26^.

Assuming stress affects functional hemispheric asymmetries does also inform the search for biological mechanisms mediating proposed relations. Therefore, two theoretical models have been formulated. (1) In the context of the negative emotionality model, Ocklenburg and colleagues^27^ discussed emotion processing under stress to drive alterations of functional hemispheric asymmetries. That is, stress induces negative affect^28^ and the right hemisphere is debated to dominate during the processing of negative affect^29^. Likewise, from an evolutionary perspective, a conserved pattern of rightward asymmetries has been proposed to get active in situations that feature novelty, unpredictability, and danger^30^—all key elements of stressors^31^. As a result, stress might prime activation of the right hemisphere. Assuming that the basal right-hemispheric priming would last some time, it would ultimately affect the processing of subsequent information. Thereby, for a period of time following stress exposure, information processing would occur under the heightened influence of the right hemisphere which might imply (a) even more dominance of the right hemisphere in tasks that it is specialized for, but also (b) a rather bilateral processing of tasks that would be dominated by the left hemisphere typically.

(2) In the context of the hormonal model, Ocklenburg and colleagues^27^ suggested that functional hemispheric asymmetries may be affected by the hormone cortisol as released under stress. Cortisol is suggested to enhance glutamatergic^32^ but to attenuate GABAergic neurotransmission^33^ via the corpus callosum—the largest white matter tract connecting the two halves of the brain (e.g.,^34^). Therefore, neurotransmission via the corpus callosum is enhanced overall. Literature still debates the nature of callosal effects on the contralateral hemisphere^34,35^. Ocklenburg and colleagues^27^ assumed the corpus callosum to exert an inhibitory influence on the contralateral hemisphere and hence predicted higher levels of cortisol in the context of stress to cause an inhibitory coupling between the two halves of the brain, which would result in stronger functional hemispheric asymmetries^27^. More precisely, increased contralateral inhibition would yield meta-control of the dominant hemisphere during a specific task at hand. Thereby, under stress, information processing may be executed even more unilaterally by the hemisphere specialized for the task at hand.

Overall, Ocklenburg and colleagues^27^ emphasized that these two theoretical models are not mutually exclusive: Cortisol and negative affect may both target functional hemispheric asymmetries under stress. Indeed, it is conceivable that the two mediators come into play at different points in time looking at the unfolding stress response. That is, negative affect is considered an immediate reaction in the face of a stressor^36^. In contrast, cortisol is released with a certain temporal delay^14^. Therefore, the negative emotionality model may explain altered functional hemispheric asymmetries in the first few minutes after stressor onset while the hormonal model may gain relevance with rising levels of cortisol in the later phase of the acute stress response.

A handful of publications tested these models in healthy individuals. It was reported that cortisol administration did not affect functional hemispheric asymmetries—either on a behavioral level^37,38^ or in neural parameters of electroencephalogram (EEG). Therefore, so far, empirical evidence has not rendered strong support for the hormonal model. Still, it is noteworthy that in given studies, behavioral tasks were not always executed at the cortisol peak of the unfolding stress response. Instead, participants completed several tasks in a row so that the different tasks partly covered slowly rising or dropping cortisol levels. In addition, tasks were mostly randomized in their order across participants. Still, one might conclude at least that cortisol does not seem capable of affecting functional hemispheric asymmetries in isolation but in interaction with further systems (e.g., negative affect). The idea of stress targeting different systems that contribute to stress-induced effects in interaction is broadly accepted in stress research^36^. Interestingly, studies reported real-life stress (i.e., TSST exposure) to partly affect interhemispheric transfer and integration of information on a behavioral level^39^. This might be interpreted as initial evidence for the idea that stress acts via agents beyond cortisol. A subsequent study, however, did not replicate this finding on a behavioral level. Still, it was shown that information transfer from the right to the language-responsive left hemisphere as assessed by means of EEG was faster after TSST exposure^40^. Therefore, one might further speculate that behavioral parameters can render null-findings while EEG measures still reveal effects on a neural level. This discrepancy indicates that on the level of the brain, functional hemispheric asymmetries may have a lower threshold concerning their reactivity to stress whereas behavioral outcomes might be less susceptible^40^. Alternatively, effects occurring on a neural level might not be transmitted to behavioral indices in a 1:1 ratio.

Berretz and colleagues^38^ finally proposed a model that integrates the results of the different studies. This model still assumes that cortisol drives effects as observed for functional hemispheric asymmetries under stress. However, it models an inverted U-curve suggesting that lateralization is only responsive to medium cortisol levels as given under real-life stress. In contrast, it was speculated that cortisol receptors may be saturated at higher cortisol levels so that observed net asymmetry is similar to non-stressful states. This idea might explain why studies did not find effects after cortisol administration where cortisol levels are substantially higher than after real-life stress. Finally, it was put forward that chronic stress rather than a single cortisol administration might have a noticeable impact.

In recent years, remote research designs have gained relevance. Concerning laterality research, this dynamic is noticeable in an app-based version (iDichotic application,^41^) of the verbal dichotic listening task on smartphones. Likewise, the verbal dichotic listening task was transferred to an online setting by Parker and colleagues^42^. The verbal dichotic listening task is an established task to assess the left-hemispheric dominance during language processing^43^. Progress in remote approaches was increased by the COVID-19 pandemic which made the use of experiments in laboratory in-person settings more challenging^44^. Indeed, in response to pandemic circumstances, also stress research adapted established stress induction paradigms to an online context. For instance, the TSST was validated for an online setting in terms of the TSST-OL^45,46^ in which the original stress protocol is realized in online communication software. Of note, since previous studies suggest that the TSST-OL produces stress reactivity of slightly smaller intensity than the original TSST^45,46^, the TSST-OL may be considered a mild stressor.

The current study seeks to elucidate open questions concerning the effects of acute stress on functional hemispheric asymmetries and makes use of an online context to this end. In detail, we examined whether exposure to the TSST-OL (henceforward referred to as an online stressor) compared to an online variant (fTSST-OL, henceforward referred to as an online control task) of the friendly TSST (fTSST,^47^) would affect functional hemispheric asymmetries as measured on a behavioral level in a healthy sample. In doing so, we focused on hemispheric dominance for language and emotion processing as well as for visuo-spatial attention. Thereby, we notably extend previous work in investigating proposed relations in terms of three different phenotypes for hemispheric dominance. Importantly, like the stress induction, the assessment of functional hemispheric asymmetries was partly implemented in online experimental software. Thereby, we further contribute to the literature on a methodological level. The study design as well as the analyses of the current project were preregistered (see “Methods” section for further details and a link to access). In this preregistration, we hypothesized that the online stressor would trigger noticeable stress reactivity in terms of subjective stress and affect markers as well as in terms of cortisol (HPA axis) and salivary alpha-amylase (sAA, SNS) compared to the online control task (set of hypotheses H1). Concerning functional hemispheric asymmetries, we hypothesized that lateralized behavioral output after the online stressor compared to the online control task would change in line with the two theoretical models. While the two theoretical models partly overlap concerning their predictions in lateralization outcomes, they associate different stress markers with alterations in functional hemispheric asymmetries. Therefore, in line with the negative emotionality model, we hypothesized differences in functional hemispheric asymmetries after the online stressor and the online control task to be mediated by negative affect (preregistered set of hypotheses H4.a). For the specific tasks, we hypothesized, a reduced advantage for right-ear input in the verbal dichotic listening task (preregistered as H4.1a), an enhanced advantage for left-ear input in the emotional dichotic listening task (preregistered as H4.2a), and an enhanced bisection error in the line bisection task (preregistered as H4.3a) after the online stressor compared to the online control task. To account for the hormonal model, alternatively, we hypothesized differences in functional hemispheric asymmetries after the online stressor and the online control task to be mediated by cortisol (preregistered set of hypotheses H4.b). Specifically, we hypothesized an enhanced advantage for right-ear input in the verbal dichotic listening task (preregistered as H4.1b), an enhanced advantage for left-ear input in the emotional dichotic listening task (preregistered as H4.2b) and an enhanced bisection error in the line bisection task (preregistered as H4.3b) after the online stressor compared to the online control task.

## Results

### Stress induction

Overall, the online stressor was successful in inducing stress in our participants. Detailed results on the subjective (negative affect, positive affect, self-reported stressfulness) and physiological (sAA, and cortisol) stress and affect markers can be found in a preprint by Heyers and colleagues^48^ while we provide a summary of these findings in section S2 of the Supplementary Material.

### Verbal and emotional dichotic listening

For the verbal and the emotional dichotic listening tasks, homonyms (one and the same syllable on both ears in the verbal dichotic listening task, one and the same emotion on both ears in the emotional dichotic listening task) can be considered as some sort of control condition. We performed three (a-c) non-parametric one-way analyses of variance (ANOVA)-like analyses using the nparLD package^49^ during which we investigated the effect of session (online stressor vs. online control task) on the sum of (a) correctly reported homonyms, (b) errors in reporting homonyms, and (c) omissions in reporting homonyms. Results as presented in Table 1 suggest that across the two sessions participants were able to discriminate the different stimuli in general.

**Table 1:** Homonyms in the verbal and in the emotional dichotic listening tasks for the online stressor and the online control task.

		Online stressor		Online control task		
		*M*	*SD*	*M*	*SD*	*p*
Verbal dichotic listening task	Correct	11.26	1.14	11.26	1.06	0.94
	Errors	0.69	1.14	0.72	1.05	0.63
	Omissions	0.05	0.22	0.02	0.14	0.26
Emotional dichotic listening task	Correct	7.57	1.72	7.66	1.65	0.64
	Errors	2.20	1.65	2.11	1.56	0.71
	Omissions	0.22	0.53	0.22	0.49	0.77

Mean, SD, and statistical comparison (*p*-value) of the number of homonyms in the verbal and in the emotional dichotic listening tasks that were identified correctly, and incorrectly and that were omitted during the two sessions (online stressor vs. online control task). Of note, the 6 homonyms were played twice during the verbal dichotic listening task, so that the maximal number of correct responses is 12. Likewise, the 5 homonyms of the emotional dichotic listening task were presented twice, so that participants may have achieved a maximal score of 10. Importantly, homonyms were randomized in order among themselves as well as among the heteronyms.

Abbreviations: M = mean, SD = standard deviation. Of note, *p*-values are not Holm-corrected.

**Fig. 1:**
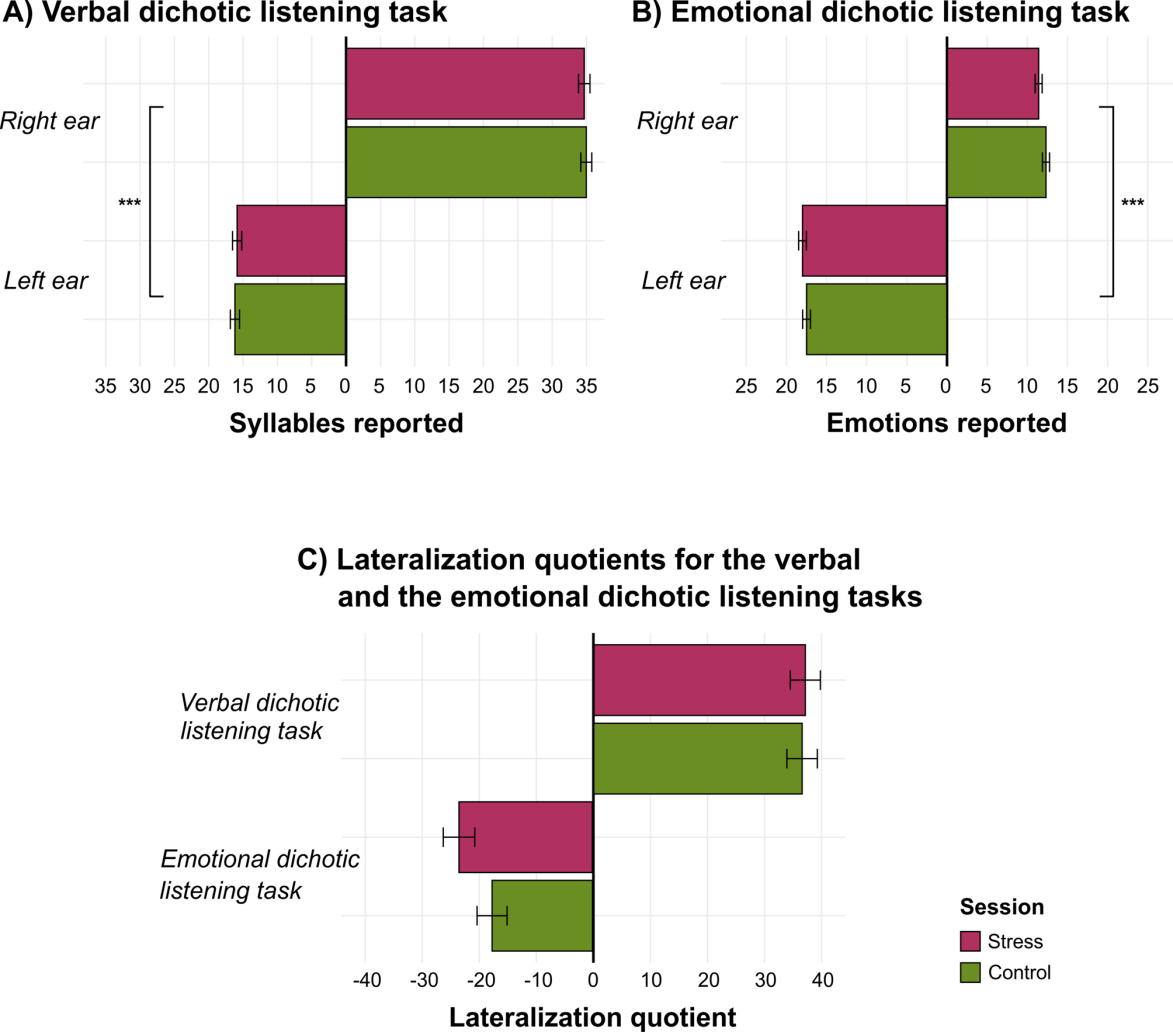
*Note* Plotted data of the verbal and the emotional dichotic listening tasks for the two sessions: online stressor (pink) and online control task (green). Panel A shows the sum of syllables reported for the two ears for presented heteronyms after the online stressor and after the online control task for *n* = 100 participants. For the verbal dichotic listening task, 30 heteronyms were presented twice to the participants so that the maximal sum per session and ear is 60. Panel B illustrates the sum of emotions reported for the two ears for presented heteronyms after the online stressor and after the online control task for *n* = 98 participants. For the emotional dichotic listening task, participants were presented with the 20 heteronyms twice so that the maximal sum per session and ear is 40. Panel C shows lateralization quotients as calculated for the verbal and the emotional dichotic listening tasks after the online stressor and after the online control task by means of the formula lateralization quotient = [(right-left)/(right + left)]*100 for *n* = 97 participants. Significant results are marked with an asterisk (*p < 0.05, **p < 0.01, ***p < 0.001). Error bars show the standard error of the mean for each bar. Of note, sample sizes do not equal the final *N* = 120 participants due to the exclusion of specified cases (see “Methods” section and the Supplementary Material for further details).

Data on heteronyms of the verbal and the emotional dichotic listening tasks are presented in Fig. 1.

Testing hypotheses on stress-induced effects (H4.1: verbal dichotic listening task H4.2: emotional dichotic listening task), we applied non-parametric ANOVA-like analysis using the nparLD package incorporating session (online stressor vs. online control task) and ear (left vs. right) as well as their interaction as factors and (a) absolute lateralization outcomes (verbal dichotic listening task: reported syllables, emotional dichotic listening task: reported emotions) or (b) relative lateralization outcomes (i.e., lateralization quotient) as dependent variables.

Considering absolute lateralization outcomes, the interaction of ear and session did not reach statistical significance either for the verbal (ATS_(1)_ = 0.01, *p*_*Holm*_ = 1.00) or for the emotional dichotic listening tasks (ATS_(1)_ = 3.15, *p*_*Holm*_ = 0.68). With respect to potential main effects, in line with that, we did not observe a main effect of session, either for the verbal (ATS_(1)_ = 2.14, *p*_*Holm*_ = 1.00) or for the emotional dichotic listening tasks (ATS_(1)_ = 0.46, *p*_*Holm*_ = 1.00). However, we found a main effect of ear for the verbal (ATS_(1)_ = 222.40, *p*_*Holm*_ < 0.001) and the emotional dichotic listening tasks (ATS_(1)_ = 83.65, *p*_*Holm*_ < 0.001). Specifically, during the verbal dichotic listening task, participants reported significantly more syllables that were presented to the right ear (*M* = 34.74, *SE* = 0.58) compared to the left ear (*M* = 15.93, *SE* = 0.47) across the two sessions. This corresponds to the typical right-ear advantage found in many studies using the verbal dichotic listening task. In contrast, during the emotional dichotic listening task, emotions were more frequently reported when they were played to the left ear (*M* = 17.62, *SE* = 0.34) compared to the right ear (*M* = 11.78, *SE* = 0.31) across the two sessions. This effect is referred to as the left-ear advantage. In line with that, in both sessions, we identified a right-ear advantage for most participants during the verbal dichotic listening task, while a left-ear advantage was observed for the majority of participants during the emotional dichotic listening task (Table 2).

**Table 2:** Right-ear advantage and left-ear advantage in the verbal and in the emotional dichotic listening tasks for the online stressor and the online control task.

	Verbal dichotic listening task		Emotional dichotic listening task	
	Online stressor	Online control task	Online stressor	Online control task
Right-ear advantage	*n* = 89 (91.75%)	*n* = 87 (89.69%)	*n* = 17 (17.53%)	*n* = 29 (29.90%)
Left-ear advantage	*n* = 8 (8.25%)	*n* = 10 (10.31%)	*n* = 80 (82.47%)	*n* = 68 (70.10%)

Number (*n*) and percentage (%) of individuals showing a right-ear advantage and a left-ear advantage in the verbal and in the emotional dichotic listening tasks separated for the two sessions (online stressor vs. online control task), respectively. A right-ear advantage was defined in terms of a positive lateralization quotient. Vice versa, a left-ear advantage equals a negative lateralization quotient in this presentation.

Analysis in terms of relative lateralization outcomes (i.e., lateralization quotient, Fig. 1, panel C) confirmed these findings. Neither for the verbal (ATS_(1)_ = 0.04, *p*_*Holm*_ = 1.00) nor for the emotional dichotic listening tasks (ATS_(1)_ = 3.38, *p*_*Holm*_ = 0.66) lateralization quotients did significantly differ after the TSST-OL and the fTSST-OL.

Regression analyses that aimed at predicting verbal (H4.1a: negative affect, H4.1b: cortisol) and emotional dichotic listening tasks (H4.2a: negative affect, H4.2b: cortisol) outcomes can be found in the Supplementary Material S3 and S4.

### Line bisection task

**Fig. 2:**
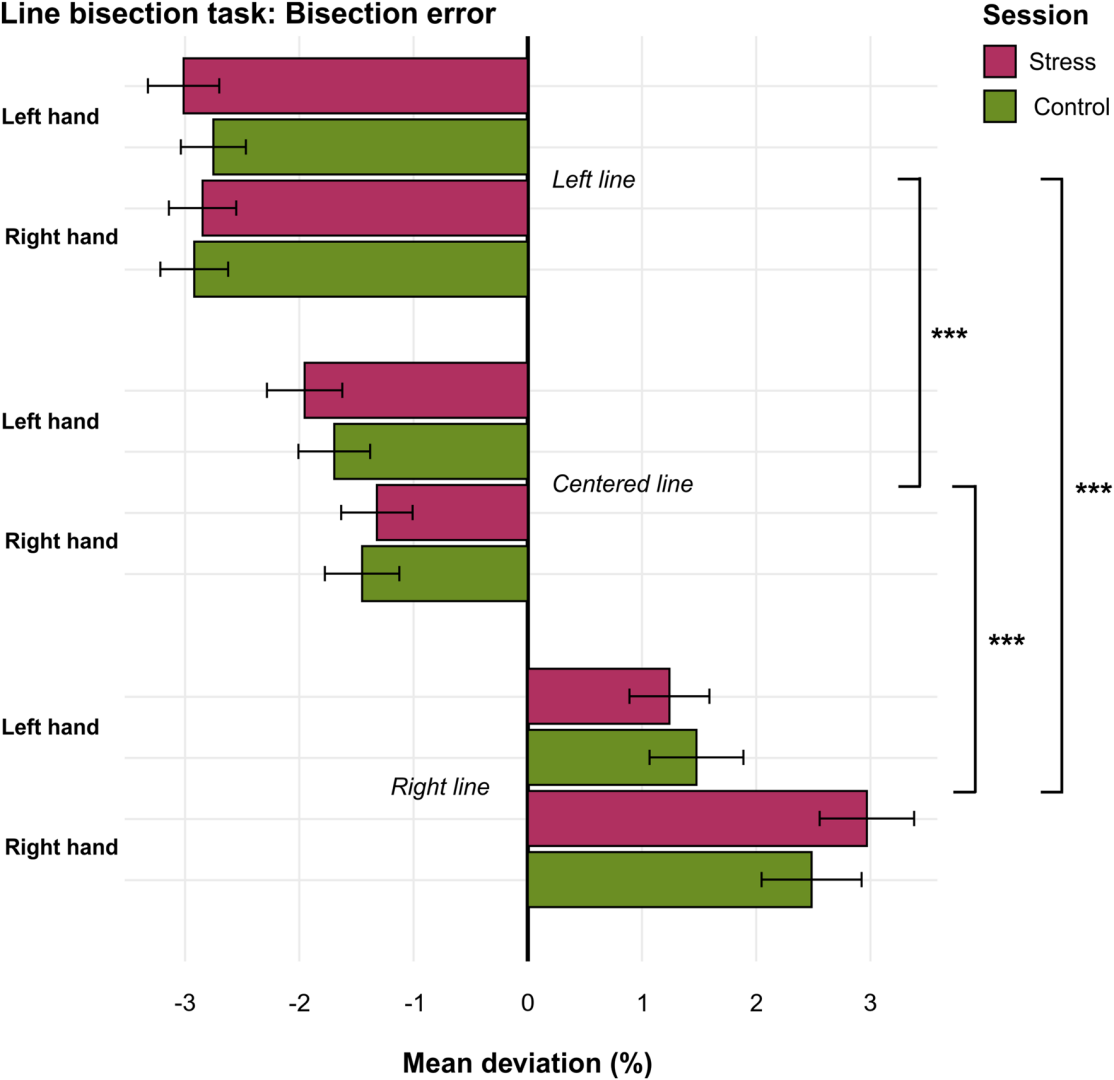
*Note* Plotted data of the line bisection task for the two sessions, online stressor (pink) and online control task (green). For each of the *N* = 17 lines, mean deviations (%) which represent the bisection error were calculated by means of the following formula: [(left half as marked by participant/veridical half)/veridical half]*100 for *n* = 115 participants. This formula calculates the percentage deviation between the midpoint as marked by the participant and the veridical midpoint of the line and weights this deviation depending on the length of the line (length of line varies between 10 and 26 cm). Lines are grouped according to their position on the original sheet where *n* = 5 lines were shifted to the left (“Left lines”), *n* = 7 were central (“Central lines”) and *n* = 5 lines were shifted to the right (“Right lines”). Participants performed the line bisection task with both hands (left hand vs. right hand) during both sessions. Significant results are marked with an asterisk (*p < 0.05, **p < 0.01, ***p < 0.001). Error bars show the standard error of the mean for each bar. Of note, sample sizes do not equal the final *N* = 120 participants due to the exclusion of specified cases (see “Methods” section and the Supplementary Material for further details).

Plotted data of the line bisection task are illustrated in Fig. 2.

Testing stress-induced effects for the line bisection task (hypothesis H4.3), we applied two separate analyses (see “Method” section for a rationale). In a first approach, we applied a non-parametric ANOVA-like analysis using the nparLD package with factors session (online stressor vs. online control task) and hand (left vs. right) as well as with their interaction. In a second approach, we repeated a non-parametric ANOVA-like analysis using the nparLD package and kept the factor session (online stressor vs. online control task) but replaced the factor hand with the factor line position (left vs. central vs. right). For both approaches, line bisection errors served as the dependent variable.

The first approach (session x hand) did not reveal a significant interaction of session and hand (ATS_(1)_ = 1.45, *p*_*Holm*_ = 1.00). Moreover, with respect to main effects, neither the factor hand (ATS_(1)_ = 5.82, *p*_*Holm*_ = 0.17) nor the factor session (ATS_(1)_ = 0.01, *p*_*Holm*_ = 1.00) did significantly affect performance during the line bisection task.

The second approach (session x line position) confirmed these results. There was no significant interaction effect of session and line position (ATS_(1.98)_ = 1.74, *p*_*Holm*_ = 1.00). Furthermore, considering potential main effects revealed the factor session did not reach statistical significance (ATS_(1)_ = 0.00, *p*_*Holm*_ = 1.00). However, the position of the line was shown to significantly affect bisection errors (ATS_(1.59)_ = 174.14, *p*_*Holm*_ < 0.001). Pairwise post-hoc tests clarified that across sessions, the bisection error significantly differed between lines shifted to the left (*M* = -5.77, *SE* = 0.35) compared to lines at a central position (*M* = -3.21, *SE* = *0.39*, *p*_*Holm*_ < 0.001) as well as compared to lines shifted to the right (*M* = 4.09, *SE* = 0.49, *p*_*Holm*_ < 0.001). Likewise, a significant difference in bisection errors was detected between central and right-shifted lines (*p*_*Holm*_ < 0.001).

Again, further regression analyses examined how far the bisection error was predicted by the different stress and affect markers (H4.3a: negative affect, H4.3b: cortisol). Results of these regression analyses are provided in the Supplementary Material S3 and S4.

## Discussion

The aim of the present study was to investigate stress-induced effects on functional hemispheric asymmetries in a remote online setting. Replicating and extending the findings of a previous study using the TSST-OL^46^ as an online stressor, we found that the TSST-OL can be applied successfully to induce psychosocial stress in terms of affect, self-reported stressfulness, sAA, and cortisol. These results are discussed in more detail in the preprint by Heyers and colleagues^48^. For the current publication, it might be noteworthy that cortisol reactivity towards our online stressor was smaller in absolute numbers when compared to in-person variants of the TSST^50^ so that the TSST-OL might be considered a mild stressor.

In order to investigate stress-induced effects on functional hemispheric asymmetries, we used online versions of the verbal and the emotional dichotic listening tasks whereas the line bisection task was assessed in paper–pencil form. Importantly, we found the expected right-ear advantage for the verbal dichotic listening task and the hypothesized left-ear advantage for the emotional dichotic listening task. Thus, our results confirm that our online adaptions of verbal and the emotional dichotic listening tasks lead to similar results as offline versions of these tasks. Of note, the verbal dichotic listening task was successfully converted to an online version^42^ as well as to a smartphone-based application (iDichotic,^41^) previously. Thus, our study replicates the findings of these previous studies with an online version of the verbal dichotic listening task and extends them by showing that the emotional dichotic listening task can also be performed online validly.

Online implementations bear the potential to increase the applicability and scope of testing batteries which might promote access to larger and more diverse samples in remote and field contexts. In this sense, a previous online version of the verbal dichotic listening task was used to investigate associations between language lateralization and motor asymmetries in a large sample of more than *N* = 600 participants^51^. Large and diverse samples were further tested with the iDichotic application to study heritability^52^ and cross-linguistic occurrence^53^ of language lateralization. Moreover, the iDichotic application facilitated research in clinical samples. For instance, after training on the dichotic listening task with the iDichotic application, Helland and colleagues^54^ observed positive effects on attention and lateralization in children with dyslexia promoting asynchronous batteries of the verbal dichotic listening task as a promising intervention tool. Of note, to the best of our knowledge, the current publication is the first study to validate an online version of the emotional dichotic listening task. As already mentioned above, we observed a left-ear advantage as the expected asymmetric outcome in our online application. Evidence favoring the validity of online versions of the emotional dichotic listening tasks might stimulate future research to study lateralization of emotion processing in similar large-scale field approaches as it was already done for language lateralization (verbal dichotic listening task). Importantly, however, the line bisection task used in the current study was applied in an established paper–pencil form. Previous studies comparing line bisection task performance between paper–pencil and computer applications indicated that different task demands of computerized versions produce divergent results (e.g.,^55,56^). Therefore, evidence suggests that not all lateralization tasks are suitable for computerized online adaption so that proper validation is required for online assessment of different laterality phenotypes. Of note, for the applied line bisection task version, we found the expected pseudoneglect which varied in strength depending on the position of the line on the sheet of paper. In particular, bisection errors were more pronounced when the line bisection task was performed for lines shifted to a leftward position^57^. In contrast to a meta-analysis of the line bisection task^57^, we did not observe an effect of hand used to bisect the line after correction for multiple comparisons was applied. Since this effect would reach significance if uncorrected, our findings imply that the effect of hand used to bisect the line on pseudoneglect is rather weak, but it may reach significance in large meta-analyses.

Concerning stress-induced effects on functional hemispheric asymmetries, the current data support null-findings as published in previous studies^37–40^ while only a few regression analyses partly rendered significant results. However, individual regression weights were rather small, suggesting that effects should be interpreted with caution. Likewise, even in joint models, the combined stress and affect markers did mostly not explain a substantial part of the observed variance. This notion is well-replicated in the laterality field. Indeed, for various laterality phenotypes, it has been shown that, even if significant, environmental predictors were of marginal importance for the overall population-based variance of that phenotype. For instance, de Kovel and colleagues^23^ using large-scale data from the UK Biobank (*N* =  ~ 500,000 participants) showed that early life factors had only a small predictive value for phenotypic handedness. Likewise, in a recent study of almost *N* = 600 participants, we did not find evidence for pre- and perinatal factors to affect laterality phenotypes such as handedness, language lateralization, and asymmetry of visuo-spatial attention^21^. As a result, our data confirm the notion that acute stress does not affect functional hemispheric asymmetries in healthy adult individuals. In this vein, our results do also not render strong support for one of the two models (negative emotionality model vs. hormonal model) as proposed by Ocklenburg and colleagues^27^ on how acute stress might affect functional hemispheric asymmetries.

The overall pattern of results as found in this study is in line with literature investigating effects on functional hemispheric asymmetries in the context of acute experimental stress induction. Indeed, previous studies did not report consistent stress-induced effects either after real-life stress exposure or after cortisol administration (e.g.,^37–40^). Of note, however, studies that included neural measures such as EEG partly reported effects suggesting that neural parameters might be more sensitive to stress exposure (e.g.,^40^). Future research might unravel how these differential thresholds are biologically embedded and under which circumstances stress would also target asymmetries on a behavioral level as it supposedly happens in clinical conditions. Moreover, looking at previous literature, it is striking that stress was revealed to rather target tasks requiring interhemispheric transfer or integration of information (e.g.,^40^). In contrast, the tasks used in the current study rather captured hemispheric dominance. This suggests that the choice of task alongside its complexity may represent an influential factor since the different tasks may assess distinct facets of the functional hemispheric asymmetries concept^58^. For instance, Berretz and colleagues^38^ speculated that cortisol would only affect functional hemispheric asymmetries as mediated via the corpus callosum so that it targets interhemispheric integration of information while hemispheric dominance is unaffected. Future studies might further unravel why interhemispheric processes and hemispheric dominance show divergent susceptibility to stress even though representing two sides of the same coin^38^. Moreover, upcoming research might dive deeper into a framework that accounts for potential differences between individuals that show approach- (e.g., anger, frustration, challenge) versus avoidance-related (fear, anxiety, threat) affective responses towards stress. In this context, previous work indicates that approach-related responses might produce a left-biased functional hemispheric asymmetry while avoidance-related responses correspond to the opposite pattern (i.e., right-biased functional hemispheric asymmetry)^59–61^.

With respect to our purpose of testing the hormonal model as proposed by Ocklenburg and colleagues^27^, it might further be relevant to consider cortisol levels in absolute numbers. That is, Berretz and colleagues^38^ put forward that functional hemispheric asymmetries may only change noticeably under moderate levels of cortisol while at lower and higher doses, there might be no effect. Indeed, as already mentioned, cortisol reactivity as measured after the current online stressor was small in absolute numbers. Therefore, cortisol secretion might not have sufficed to produce noticeable effects on functional hemispheric asymmetries. Last but not least, it is noteworthy that the remote online context may have affected the data collected in the current study. Even though we confirmed the validity of our online assessment of the verbal and the emotional dichotic listening tasks, we cannot fully rule out that remote online contexts feature more general characteristics that subtly prime a lateralized involvement of the two hemispheres that deviates from real-world scenarios. Still, in sum, the current pattern of results indicates that functional hemispheric asymmetries do not change flexibly in response to acute stress exposure in adult healthy individuals but that they represent a rather stable trait.

This study does not come without limitations. First, lateralization tasks were executed in randomized order so that the exact temporal delay between each lateralization task and the online stressor or the online control task onset varied across participants. This implies that the different lateralization tasks were not necessarily executed at the peak level of the various stress and affect markers. Indeed, since we incorporated a great range of stress and affect markers, we were forced to prioritize one of these and spread lateralization tasks around this single marker’s peak level. We opted to focus on cortisol in order to test the hormonal model as proposed by Ocklenburg and colleagues^27^. However, that way, we were probably not able to evaluate effects on functional hemispheric asymmetries as mediated via stress-induced changes in subjective markers as well as in sAA as these rather occur shortly after stressor onset (e.g.,^62,63^). As a result, we did not truly cover the negative emotionality model as put forward by Ocklenburg and colleagues^27^. Future studies investigating the two theoretical models might consider giving one and the same lateralization task at different points in time across the unfolding stress response. Thereby, it might be possible to capture effects as induced by different stress and affect markers. However, such a design might be confounded by training or exhaustion effects. Second, with respect to the verbal and the emotional dichotic listening tasks, participants of the current study partly used their own headphones during task completion. To ensure the validity of the listening tasks, we checked that the headphones used by participants were functional on both ears. Additionally, we implemented an audiometer task which controlled for the participants’ hearing ability at both ears as well as for the headphones’ functionality at different frequencies. Since the verbal and the emotional dichotic listening tasks produced the expected asymmetric effects, it is conceivable that the headphones used by participants were adequate. Third, since online variants of the verbal and the emotional dichotic listening tasks were not executed twice under non-stressful conditions, we cannot provide test–retest reliability estimates. Of note, for online versions of the verbal dichotic listening task, previous studies did include such indices and thereby suggested satisfying reliability^42^. Therefore, we assume that our online battery was comparably reliable and valid. Fourth, our study may be limited by the fact that we only included right-handed participants. Indeed, greater variability in trait lateralization (e.g., left-handedness) might have unraveled further effects. Along these lines, our sample might not be considered representative of a general population. Fifth, non-effects of the current study might also query the appropriateness of included laterality phenotypes to capture environmental influences on functional hemispheric asymmetries. Indeed, as we argued in a previous publication^64^, social laterality phenotypes might better serve for unraveling environmental determinants of lateralization since they underlie stronger evolutionary pressure. However, social laterality phenotypes might be more difficult to assess in online test batteries. In line with that, sixth, we were not able to include neural measures into our online battery. Of note, previous studies showed that stress-induced effects on functional hemispheric asymmetries might be better observed in EEG correlates, for instance, than in behavioral parameters^40^. Interestingly, existing literature already validated mobile EEG applications that allow measurement in field contexts (for a review, see^65^). However, the current study was partly realized in a true remote setting where there was no in-person encounter between participant and experimenter and where testing material was partly only provided via postal delivery. Therefore, for the current design, even mobile applications of neural measurement would not have been suitable. As a result, during the current design, we rather prioritized the comprehensiveness of our remote online approach even though this was at the cost of neural measures. The adequacy of remote online designs must consequently be evaluated in light of individual research questions for future projects.

To conclude, the current study was successful in further validating online adaptions of established lateralization tasks (i.e., verbal and emotional dichotic listening tasks) and thus promotes future investigations of language- and emotion-related laterality phenotypes in remote large-scale approaches. In the same vein, the current study still emphasizes that not all laterality phenotypes might serve equally well for online assessment. For instance, we opted against an online-based evaluation of lateralized visuo-spatial attention by means of the line bisection task and thus recommend individual validation of online assessment for different laterality phenotypes. With respect to stress-induced effects on functional hemispheric asymmetries, the current study confirmed previous literature in rather rendering null-findings. Consequently, functional hemispheric asymmetries in adult healthy individuals seem to represent a relatively stable trait that does not fluctuate strongly in response to acute temporally limited stress exposure of milder intensity. Of note, future research might entangle whether stress as experienced earlier in life does modulate functional hemispheric asymmetries and structural lateralization and how such stress exposure might coincidence with clinical outcomes.

## Methods

The data analysis of the current study was preregistered at the Open Science Framework (OSF) under the link https://osf.io/q6tn7/?view_only=76aa6bb5bc794ca1bc3d42147f48f542. Specifically, the preregistration was drafted in the sense of a secondary data analysis which was uploaded at a point in time where data collection of the current study was already running but before any inspection or analysis of the data had taken place. From this preregistration, it is evident that the current study was part of a larger project of which a preprint has already been published elsewhere (Heyers and colleagues,^48^). In this existing publication, Heyers and colleagues^48^ dealt with the preregistered sets of hypotheses H1, H2, and H3. The preregistered set of hypotheses H1 covered a further validation of the TSST-OL (online stressor) compared to the fTSST-OL (online control task) (session represented a within-subjects factor as all participants underwent the online stressor as well as the online control task in randomized order). Specifically, for a successful validation of the online stressor, the preregistered set of hypotheses H1 expected the online stressor to produce decreased positive affect, increased negative affect, increased self-reported stressfulness, increased cortisol levels, and increased sAA activity as compared to the online control task. The preregistered set of hypotheses H2, which was also addressed by Heyers and colleagues^48^, further expected that successful stress induction using the online stressor may depend on the application context (home-based application vs. laboratory-based application). Finally, in line with the preregistered set of hypotheses H3, Heyers and colleagues^48^ assessed stress-induced effects on state empathy performance measures. A short summary of the results presented by Heyers and colleagues^48^ can be found in the Supplementary Material (section S2). Importantly, the publication by Heyers and colleagues^48^ did not include results on stress-induced effects on functional hemispheric asymmetries (preregistered set of hypotheses H4). Thus, stress-induced effects on functional hemispheric asymmetries in line with the preregistered set of hypotheses H4 are the focus of the current publication. That is, beyond the empathy task, in the referenced study, participants further completed three tasks assessing functional hemispheric asymmetries after exposure to the online stressor and the online control task. Since empathy and hemispheric asymmetries are largely unrelated topics, a separation into two manuscripts is important in order to avoid an unnecessarily complicated and long paper.

### Sample

Recruitment of the current sample took place via online advertisements and local announcements. In total, we tested *N* = 120 (*n* = 60 women) healthy volunteers (see Heyers and colleagues^48^ for our sample size calculation) so that our sample is not representative of a general population. Participants were of normal weight (body mass index, BMI: *M* = 22.48 kg/m^2^, *SD* = 1.96 kg/m^2^) and aged *M* = 24.03 (*SD* = 4.20) years. Handedness of participants was assessed in advance by means of the Edinburgh Handedness Inventory (EHI,^66^) which accounts for a lateralization quotient by means of the formula

$${\text{lateralization quotient }} = \, \left[ {\left( {{\text{right}} - {\text{left}}} \right)/\left( {{\text{right}} + {\text{left}}} \right)} \right]*{1}00$$

We only included right-handed participants with an EHI lateralization quotient ≥ 60. Finally, the average EHI lateralization quotient of the total sample was *M* = 88.99 (*SD* = 12.12).

Furthermore, participants must not report any history or current presence of mental or physical disorders. Likewise, current extraordinary psychological stress as well as previous participation in experiments on psychosocial stress were considered exclusion criteria. In general, against the background of our experimental stress induction, we tried to control factors that can potentially disrupt HPA axis reactivity. As a result, participants must not report regular consumption of drugs or medication, and recent jetlag, vaccination, blood donation, and cold or viral infections. Psychology students participating in the current study must not be more advanced than the 3rd semester. Women did not take any hormonal contraceptives and we ensured that both testing sessions were scheduled during the same phase of the menstrual cycle (follicular phase vs. luteal phase). Finally, participants tested from home were further screened for technical prerequisites (see Heyers and colleagues,^48^, for further details on the context manipulation).

This study was approved by the local ethics committee of the Faculty of Psychology at the Ruhr University Bochum and was conducted in accordance with the Declaration of Helsinki. In line with that, all participants gave informed consent. Data collection took place between 04/2021 and 11/2023 at research facilities of the Ruhr University Bochum, Germany, or at the participants’ homes.

### Experimental procedure

The experimental procedure is described in the above-mentioned preregistration as well as in the preprint by Heyers and colleagues^48^ in full length. Therefore, in the following, we will only elaborate on such details of the experimental procedure that are relevant to the current manuscript. That is, participants were invited to two testing sessions in a within-subjects design. During these two sessions, participants underwent the online stressor as well as the non-stressful online control task. The order in which participants were exposed to the online stressor and the online control task (1. Online stressor—2. Online control task vs. 1. Online control task—2. Online stressor) was randomized across participants but balanced across the whole sample. The online stressor (TSST-OL) was introduced by Gunnar and colleagues^45^ as an online variant of the well-established TSST. That is, like in the TSST, participants undergo a preparation phase and a speech part plus a math part in front of a non-responsive, social-evaluative panel (one male, one female researcher in white laboratory coats). Indeed, during the TSST-OL, this procedure is realized in online communication software (Zoom™, https://www.zoom.us). In the publication by Heyers and colleagues^48^, we further validated the TSST-OL for an adult population. Moreover, we further investigated the relevance of the application context. That is, half of the participants completed the two testing sessions from home while the other half were seated in a laboratory room (see Heyers and colleagues,^48^, for more details).

Concerning the online stressor, we adhered to the standard protocol as applied in the in-person TSST and in the so-far existing TSST-OLs. The success of the stress manipulation was assessed through different stress parameters as sampled across exposure to the online stressor and the online control task. In detail, participants completed the Positive and Negative Affect Schedule (PANAS, German version:^67^) as well as a visual analogue scale (VAS, as adapted from^68^) which assessed the perceived stressfulness of the previous situation (i.e., participants evaluated the stressfulness of the previous situation on a scale from 0 (“not stressful at all”) to 100 (“maximally stressful”)). In parallel, participants gave saliva samples that were analyzed for cortisol (as a marker for HPA axis reactivity) as well as for sAA activity (as a marker for sympathetic activity^69^). These stress markers were collected at − 26 min. (T1), -6 min. (T2), + 7 min. (T3), + 26 min. (T4) and + 56 min. (T5) relative to stressor or control onset. After exposure to the online stressor and the online control task, participants finally engaged in an empathy task and in three lateralization tasks in randomized order.

### Assessment of functional hemispheric asymmetries

**Fig. 3:**
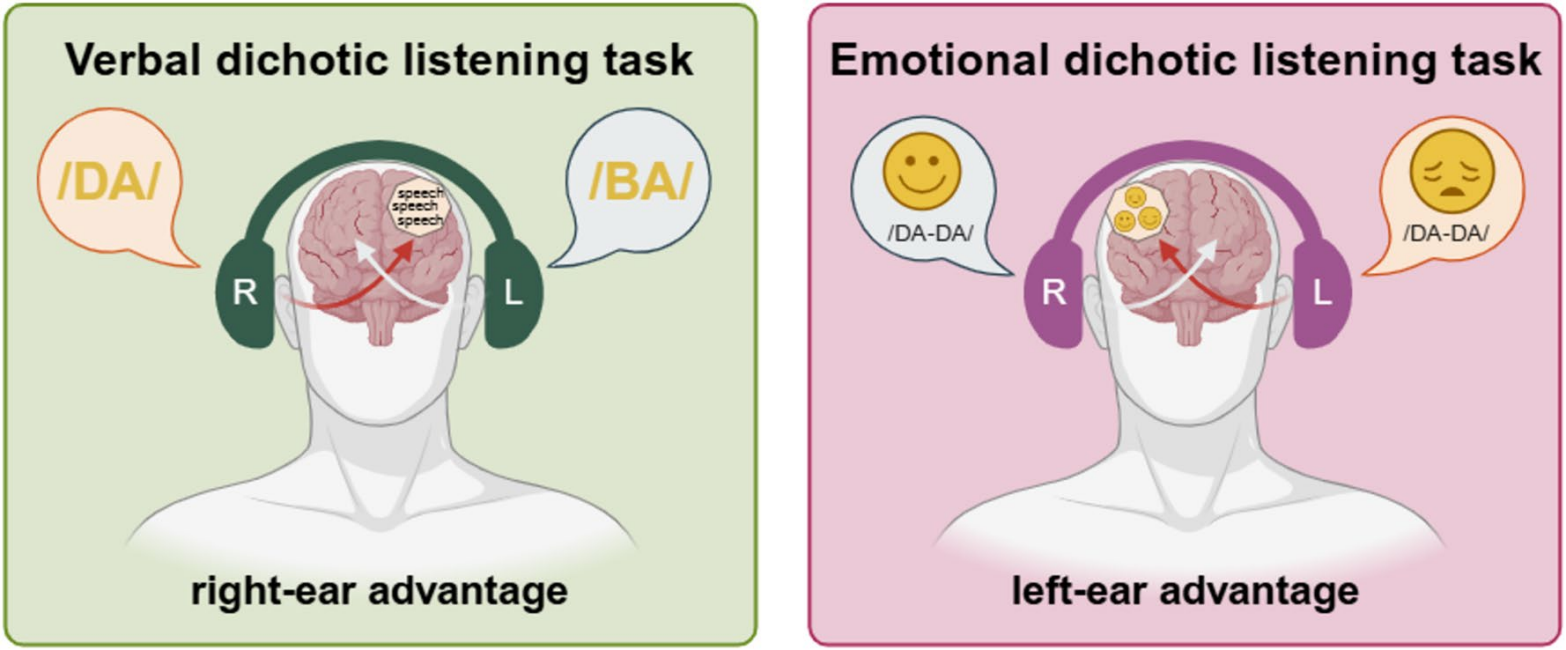
*Note* Schematic illustration of the verbal (left) and the emotional dichotic listening tasks (right). During the verbal dichotic listening task, due to the crossing of auditory tracts, verbal input (i.e., syllables) of the right ear is more quickly transferred to the left hemisphere while input from the right ear is initially registered by the right hemisphere. As a result, left-hemispheric language areas can more quickly process input from the right ear which leads to the so-called right-ear advantage. During the emotional dichotic listening task, the crossing of auditory tracts evokes the same pattern of crossed information receipt. However, stimuli in the emotional dichotic listening task are of emotional quality and thus get processed more quickly by the right hemisphere resulting in the so-called left-ear advantage. This figure was created at Biorender.com.

**Fig. 4:**
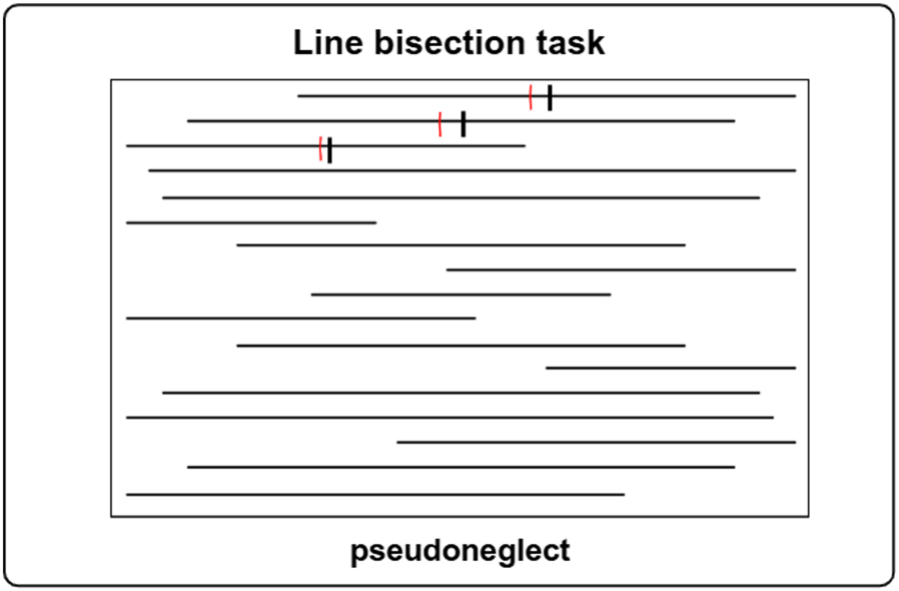
*Note* Schematic illustration of the line bisection task. Exemplary, for the first three lines, the figure shows the veridical middle of each line (black) as well as a fictive marked half (red). The fictive marked half is simulated to rather deviate to the left side of the veridical half for these fictive data. Indeed, deviations to the left of the veridical half are in line with the expected pseudoneglect. This figure was created at Biorender.com.

To measure functional hemispheric asymmetries, participants completed three lateralization tasks after the online stressor and the online control task. In detail, participants completed a verbal and an emotional dichotic listening task (Fig. 3), as well as a line bisection task (Fig. 4). The order of these tasks was randomized across participants but kept the same for one participant across the two sessions. The verbal and the emotional dichotic listening tasks were programmed using PsychoPy^70^ and run online using Pavlovia experimental software (https://pavlovia.org/) while the line bisection task was performed in paper–pencil format. Of note, in line with open and reproducible science practices, the verbal and the emotional dichotic listening tasks have been made publicly available for open usage in the OSF project of the current study (link: https://osf.io/q6tn7/?view_only=76aa6bb5bc794ca1bc3d42147f48f542).

The verbal dichotic listening task of the current study was in line with the standard Bergen application^43^ and followed recommendations by Westerhausen and colleagues^71^. Participants were presented with six syllables (/BA/, /DA/, /GA/, /KA/, /PA/, /TA/) in dichotic stereo mode so that one syllable was presented to the left ear while a second syllable was presented simultaneously to the right ear. Participants were asked to report which syllables they heard best. This was done by pressing a specified key on their keyboard (1–6). To press the keys, participants were instructed to either use their left or their right hand at the beginning of the verbal dichotic listening task. The hand to use was randomized and then switched right in the middle of the paradigm. Response time was fixed to a duration of 3000 ms whereas inter-trial intervals were of varying length (500 ms—1000 ms)^71^. Twelve practice trials were completed at the beginning of the verbal dichotic listening task. Practice trials covered the dual presentation of six homonyms. In the test phase, participants were confronted with all possible combinations of syllables (36 combinations: six homonyms and 30 heteronyms) in two runs. The verbal dichotic listening task took approximately 8 min. to complete. On average, the verbal dichotic listening task was performed + 38 min. after the onset of the online stressor or the online control task.

During the emotional dichotic listening task, participants heard only one syllable (/DA-DA/) on both ears. However, the syllable was presented with different emotional intonation to the left and the right ear. In total, there were five emotional intonations (/happy/, /sad/, /surprised/, /angry/, /neutral/). Auditory stimuli were taken from Hahn and colleagues^72^. Participants were asked to report which emotional intonation they heard best. Again, this was done using the keyboard and pressing the specified key (1–5) either with the left or the right hand while the hand to use was changed between the two runs of the emotional dichotic listening task. Response time and inter-trial intervals were of the same length as in the verbal dichotic listening task. The emotional dichotic listening task started with ten practice trials which covered the dual presentation of the five homonyms. The test phase consisted of two runs each involving the presentation of all combinations of emotional intonations (25 combinations: five homonyms and 20 heteronyms). Participants required approximately 6 min. to complete the emotional dichotic listening task. On average, the emotional dichotic listening was performed + 38 min. after the onset of the online stressor or the online control task.

The line bisection task^57^ involved the bisection of several lines at a perceived midpoint. The line bisection task was performed with the left and the right hand on two different sheets of paper. That is, for one sheet, participants were instructed to hold their pen in the left hand while bisecting the lines, while the pen was switched to the right hand to complete the other sheet. Importantly, we randomized the hand to use first across participants. The sheets completed with the left and the right hand were totally identical in size (A4 size, 21.0 cm × 29.7 cm) and makeup. They both consisted of 17 horizontal lines of different lengths and varying positions. In detail, the length of the lines varied between 10.0 cm and 26.0 cm while all of them were 1 mm in width. Seven lines were positioned right at the center of the sheets whereas five of them were shifted to the left and to the right margin, respectively. Participants were instructed to mark the midpoint of each line using a vertical dash bisecting the lines. There was no time limit to complete the task. However, participants were instructed to bisect the lines intuitively not spending too much cognitive effort. Importantly, participants were required to only rely on their subjective sense of proportion. Accordingly, it was forbidden to use any tools (e.g., a ruler) or to use the hands for gauging the lines. We instructed participants to sit in an upright position and to place the sheets in front of them in a parallel manner. At the beginning of the first session, participants had the opportunity to try out the line bisection task on four lines with their preferred hand. Participants required approximately 5 min. to complete the line bisection task. On average, the line bisection task was performed + 41 min. after the onset of the online stressor or the online control task.

### Audiometer

Importantly, for the verbal and emotional dichotic listening tasks, we aimed to ensure that participants have normal hearing ability at both ears and no pronounced hearing asymmetries between the ears. Therefore, participants engaged in an audiometer test at the beginning of the second session (irrespective of whether the second session was their stress or control session). In detail, we applied an online audiometer test (https://www.audiocheck.net/testtones_hearingtestaudiogram.php) during which we presented several tones at different frequencies (8000 Hz, 4000 Hz, 2000 Hz, 1000 Hz, 500 Hz) with increasing volume. At each volume, participants were instructed to indicate whether they heard a tone or not. With that, we aimed to identify the threshold (volume) at which participants could certainly perceive a tone for the different frequencies. Importantly, this procedure was executed for the left and the right ear separately. Participants whose hearing threshold differed more than 15 dB between the left and the right ear at one of the presented frequencies, were excluded from the analysis of verbal and emotional dichotic listening tasks (see section S1 in the Supplementary Material for further details).

### Statistical analysis

Data handling was realized in R (version 4.3.2) implementation in RStudio^73^. Data analysis was preregistered at OSF and can be accessed via the link https://osf.io/q6tn7/?view_only=76aa6bb5bc794ca1bc3d42147f48f542. In this OSF project, we also uploaded data as well as analysis scripts.

For the verbal and the emotional dichotic listening tasks, we calculated lateralization quotients according to the formula that was already introduced above (lateralization quotient = [(right-left)/(right + left)]*100). In this formula “right” corresponds to syllables (verbal dichotic listening task)/emotions (emotional dichotic listening task) that were correctly chosen for the right ear during the presentation of heteronyms while “left” corresponds to syllables (verbal dichotic listening task)/emotions (emotional dichotic listening task) that were correctly identified for the left ear during the presentation of heteronyms (syllables that were identified incorrectly or cases in which an answer was omitted, were not taken into account for this calculation). For the line bisection task, we measured deviations between the perceived midpoint (as marked by the participants) and the veridical midpoint of the line. Since the different lines varied in absolute length, we relativized deviations using the formula [(left half as marked by participant/veridical half)/veridical half]*100. For stress measures, we used area under the curve (AUC) measures according to formulas provided by Prüssner and colleagues^74^ which were calculated already for the publication by Heyers and colleagues^48^ (see there for further details).

Prior to statistical analysis, we performed an outlier exclusion. Outliers were defined as values that deviated more than three standard deviations (SD) from the mean of the respective measure. Outlier exclusion was performed for the different dependent variables separately, so that analyses for verbal and emotional dichotic listening tasks, lateralization quotients, and line bisection task may cover different samples. Moreover, and as already specified above, for the verbal and emotional dichotic listening tasks, we further excluded participants who showed divergent hearing ability between the left and the right ear exceeding 15 dB in the audiometer test. Section S1 of the Supplementary Material specifies how many individuals were excluded on the basis of these criteria for the different lateralization tasks.

Subsequently, we checked the normality and homogeneity of variance of our data as required by the preregistered ANOVA and regression analyses. In detail, we applied the Shapiro–Wilk test as well as visual inspection of QQ-plots to assess the normality of data for ANOVA. Levene’s tests were utilized to test the homogeneity of variance. For regression analyses, we applied visual inspection methods. In general, statistical assumptions were checked for data from the lateralization tasks. In contrast, data on stress markers were already covered by Heyers and colleagues^48^. In line with that, we did not integrate the context manipulation (home-based vs. laboratory-based testing) into our analyses of the current publication. That is, because we did not have any hypotheses concerning the context manipulation with respect to asymmetry-related research questions (also see our preregistration). However, a comprehensive analysis incorporating the factor of testing context can be found in the preprint by Heyers and colleagues^48^.

In line with our preregistered set of hypotheses H4, for the verbal and the emotional dichotic listening tasks, we aimed to investigate the occurrence of expected lateralization effects (in terms of a manipulation check) as well as stress-induced effects. In line with our preregistration, the set of hypotheses H4 was tested using parametric ANOVA in cases where data fulfilled statistical assumptions while we opted for a non-parametric alternative in terms of the nparLD-package^49^ whenever statistical assumptions were violated. In line with that, we report ANOVA-type statistics (ATS). During this analysis, behavioral lateralization indices were operationalized in terms of two approaches: (1) In the first approach, we modeled absolute lateralization outcomes such as the absolute number of syllables (verbal dichotic listening task) and emotions (emotional dichotic listening task) as reported for the left and the right ear as a function of ear (left vs. right) and session (online stressor vs. online control task) or their interaction (ear x session). (2) In a second approach, we modeled relative, aggregated lateralization outcomes (i.e., lateralization quotients for the verbal and the emotional dichotic listening tasks as calculated by the formula provided above) as a function of session (online stressor vs. online control task). Of note, for the line bisection task, we had to deviate from the preregistered analysis. We originally planned to run a model with three within-factors including hand (left vs. right), line position (left vs. centered vs. right), and session (online stressor vs. online control task). However, since line bisection task data did not fulfill the statistical assumptions of ANOVA, we used the specified nparLD-package^49^ which cannot handle three within-factors simultaneously. Therefore, we included the different independent factors in separate models. That is, first, we modeled the bisection error as a function of session and hand as well as their interaction (session x hand). Second, we investigated how far session and line position or their interaction (session x line position) were predictive of bisection errors.

Our preregistered set of hypotheses H4 did not only involve the investigation of pure occurrence of stress-induced effects in behavioral lateralization outcomes. Instead, hypotheses made assumptions on specific stress markers driving supposed effects. In particular, the preregistered set of hypotheses H4a assumed negative affect to drive stress-induced effects on functional hemispheric asymmetries. In contrast, the preregistered set of hypotheses H4b suggested cortisol to produce stress-induced effects on functional hemispheric asymmetries. In order to test these assumptions, we used multiple linear regression models with AUC with respect to increase (AUCi) measures of negative affect as given by the PANAS (preregistered set of hypotheses H4a) or cortisol (preregistered set of hypotheses H4b) as predictors. In turn, lateralization outcomes for the verbal dichotic listening task (syllables reported per ear and lateralization quotient), the emotional dichotic listening task (emotions per ear and lateralization quotient), and the line bisection task were included as outcomes. Finally, on an exploratory basis, we also included AUCi measures with the remaining stress and affect markers. That is, we also included positive affect as given by the PANAS, VAS ratings reflecting self-reported stressfulness, and sAA. Of note, all different AUCi measures were included as predictors simultaneously in the multiple linear regression models. We ran regressions across the online stressor and the online control task as well as for the online stressor alone.

As a significance level, we used α = 0.05 after Holm-correction for multiple testing. In order to further unravel significant main and interaction effects, we performed pairwise post-hoc tests, also with Holm-correction.

## Supplementary Information

Supplementary Tables.

## Data Availability

The preregistration, raw data and R scripts as well as the dichotic listening tasks of the current publication can be retrieved from OSF under the link https://osf.io/q6tn7/?view_only=76aa6bb5bc794ca1bc3d42147f48f542.
