# Supplementary material for: Using the online version of the Trier Social Stress Test to investigate the effect of acute stress on functional lateralization
Source: Sci Rep. 2024 Sep 6;14:20826. doi: 10.1038/s41598-024-71668-w (PMC11379872; doi:10.1038/s41598-024-71668-w)
Supplement: Supplementary file 1 — Supplementary Tables. [file 41598_2024_71668_MOESM1_ESM.docx]

- Supplementary Material

**Using the online version of the Trier Social Stress Test to investigate the effect of acute stress on functional lateralization**

**S1 Exclusion: Audiometer and outliers**

For the verbal and the emotional dichotic listening tasks, *n*=16 participants were excluded from the datasets due to the audiometer test. Concerning exclusions based on the three SD-criterion (see main manuscript), for the verbal dichotic listening task as performed after the online stressor, no participant was identified as an outlier. Instead, in the data on the verbal dichotic listening task as gathered after the online control task, *n*=1 participant was identified as an outlier for syllables as reported for the left ear, the right ear as well as for the lateralization quotient as an aggregated measure, respectively. For the emotional dichotic listening task, *n*=3 participants were identified as outliers for syllables as reported for the left ear after the online stressor. After the online control task, we identified *n*=1 participant for syllables as reported for the left ear, the right ear as well as for the lateralization quotient as an aggregated measure, respectively. Finally, for the line bisection task as performed after the online stressor, we identified *n*=1 outlier for lines at a central position as bisected with the left hand and *n*=1 outlier for lines shifted to the right as bisected with the right hand, respectively. *n*=2 participants were further identified as outliers for lines shifted to the left as bisected with the left hand in the line bisection task after the online control task.

Since some of the outliers overlapped between the different conditions, exclusions of participants identified as outliers and as identified by means of the audiometer let to the following sample sizes: *n*=100 participants were available for the analysis of syllables as reported for the left and the right ear during the verbal dichotic listening task after the two sessions. In parallel, the dataset concerning emotions as reported for the left and the right ear in the emotional dichotic listening task after the two sessions covered *n*=98 participants. For session-dependent analysis of the lateralization quotient in the verbal and in the emotional dichotic listening tasks, we were able to include *N*=97 participants. Last but not least, *n*=115 participants were included in the analysis of the line bisection task as performed with the two hands, the three types of line position and the two sessions.

**S2 Stress induction: Stress and affect markers**

To summarize the results reported by Heyers and colleagues [47], a main effect of session showed that compared to the online control task, during the online stressor participants reported lower positive affect (PANAS, *p*<.05) but higher negative affect (PANAS, *p*<.001) and higher VAS ratings of stressfulness (*p*<.001). Moreover, during the online stressor, we found higher cortisol levels (*p*<.05). Of note, however, there was no difference in sAA activity between the online stressor and the online control task (*p*=.13). Moreover, we found significant interaction effects of session (online stressor vs. online control task) and time (T1 vs. T2 vs. T3 vs. T4 vs. T5) for positive affect (PANAS, *p*<.001), negative affect (PANAS, *p*<.001), self-reported stressfulness (VAS, *p*<.001), cortisol levels (*p*<.001) and sAA activity (*p<*.05). Pairwise post-hoc comparisons further confirmed that the differences between the online stressor and the online control task occurred at the expected points in time so that effects were interpreted as stress-induced for all measures.

**S3 Results of regression analyses**

*Verbal and emotional dichotic listening tasks*

Regression analyses that aimed at predicting verbal (H4.1a: negative affect, H4.1b: cortisol) and emotional dichotic listening task (H4.2a: negative affect, H4.2b: cortisol) outcomes across both sessions replicated the null-findings of previous analyses. For the verbal dichotic listening task, neither syllables as reported for the left (*p*=.36, see Table S4.1) nor the right ear (*p*=.39, see Table S4.2) were significantly predicted by the different stress and affect markers collectively. For the emotional dichotic listening task, concerning emotions as reported for the left (*p*=.14, Table see Table S4.7) as well as for the right ear (*p*=.48, see Table S4.8), the multiple stress and affect markers had no significant predictive value overall. However, for emotions as reported for the left ear, AUCi of positive affect seemed to be of predictive value individually (β=0.04, t=2.09, *p*<.05). This effect indicated that emotions as reported for the left ear during the emotional dichotic listening task increased with increasing positive affect. Last but not least, lateralization quotients of the verbal (*p*=.29, see Table S4.3) and the emotional dichotic listening tasks (*p*=.24, see Table S4.9) were not significantly predicted by means of the overall regression model. However, lateralization quotients for the verbal dichotic listening task were individually predicted by AUCi of cortisol (β=0.06, t=2.06, *p*<.05) in that lateralization quotients of the verbal dichotic listening task seemed to rise with increasing AUCi of cortisol.

Considering the online stressor in isolation, replicated this pattern of results. For the verbal dichotic listening task, overall regression models were not of predictive for syllables reported for the left (*p*=.27, see Table S4.4) or the right ear (*p*=.65, see Table S4.5). Likewise, emotions as reported during the emotional dichotic listening task for the left (*p*=.86, see Table S4.10) and the right ear (*p*=.75, see Table S4.11) were not predicted by the combined stress and affect markers in the stress session only. Finally, neither lateralization quotients of the verbal (*p*=.37, see Table S4.6) nor the emotional dichotic listening tasks (*p*=.63, see Table S4.12) showed significant associations with the regression model of combined stress and affect markers during the stress session.

*Line bisection task*

Again, regression analyses examined in how far the bisection error was predicted by the different stress and affect markers (H4.3a: negative affect, H4.3b: cortisol) across the two sessions. Bisection errors as produced with the left hand were not predicted by the various predictors collectively for lines at different positions (left: *p*=.27, see Table S4.13, right: *p*=.70, see Table S4.15, central: *p*=.05, see Table S4.14). However, AUCi of negative affect (PANAS) (β=-0.06, t=-3.15, *p*<.01) as well as AUCi of self-reported stressfulness (VAS) (β=0.00, t=2.63, *p*<.01) contributed to the bisection error for central lines as bisected with the left hand individually. These relations suggested that bisection errors for central lines were smaller when negative affect increased but larger with heightened self-reported stressfulness. Performance during the line bisection task with the right hand yielded a similar pattern of results in that the overall regression model was not predictive for lines at a central position (*p*=.74, see Table S4.17) and for lines shifted to the right (*p*=.74, see Table S4.18). However, even though lines shifted to the left and bisected with the right hand were not predicted by the combined stress and affect markers (*p*=.12, see Table S4.16), AUCi of positive affect (PANAS) was revealed to have a statistically significant negative effect (β=-0.03, t=-2.28, *p*<.05) so that bisection errors reduced with increasing negative affect.

Considering bisection errors during the online stressor only, rendered a similar picture. Together, the different stress and affect markers could not significantly predict performance during the line bisection task as shown with the left hand for lines shifted to the left (*p*=.26, see Table S4.19). However, individually, this condition was predicted by AUCi of sAA (β=0.00, t=2.14, *p*<.05) suggesting higher pseudoneglect with increasing sAA activity. The overall model was also not associated with bisection performance with the left hand for lines at a central (*p*=.28, see Table S4.20) or right-shifted position (*p*=.57, see Table S4.21). Still, individually, AUCi of negative affect (PANAS) was shown to have a significant effect for central lines bisected with the left hand (β=-0.05, t=-2.40, *p*<.05) suggesting that for this condition, increases in negative affect were associated with decreases in bisection errors. Finally, bisection errors as produced with the right hand after the online stressor, were not explained by the overall regression model for left-shifted (*p*=.06, see Table S4.22), central (*p*=.06, see Table S4.23), or right-shifted lines (*p*=.80, see Table S4.24). However, lines shifted to the left and bisected with the right hand after the online stressor were individually predicted by AUCi of positive affect (PANAS) (β=-0.03, t=-2.04, *p*<.05) and self-reported stressfulness (VAS) (β=-0.00, t=-2.20, *p*<.05) with bisection errors suggested to decrease with increasing levels of these markers.

**S4 Comprehensive outputs of regression analyses**

**Table S4.1**

**Regression model on syllables as reported for the left ear during the verbal dichotic listening task across both sessions.**

| **Coefficients** | **Estimate** | **Std. Error** | **t value** | **Pr(>\|t\|)** |
| --- | --- | --- | --- | --- |
| (Intercept) | 15.46 | 0.96 | 16.155 | < 0.001*** |
| Positive affect (PANAS) | -0.02 | 0.03 | -0.830 | 0.4080 |
| Negative affect (PANAS) | -0.04 | 0.05 | -0.980 | 0.3292 |
| Self-reported stressfulness (VAS) | 0.00 | 0.00 | 1.379 | 0.1705 |
| Cortisol | -0.01 | 0.01 | -1.687 | 0.0941 |
| sAA | 0.00 | 0.00 | 0.289 | 0.7733 |

*Note.* Table shows statistical parameters for a linear multiple regression model with syllables as reported for the left ear during the verbal dichotic listening task as outcome and stress and affect markers as predictors. Regression model was run across both sessions (online stressor and online control task). Stress and affect markers were included in form of AUCis. Signif. codes: 0 '***' 0.001 '**' 0.01 '*' 0.05 '.' 0.1 ' ' 1. Residual standard error: 6.887 on 122 degrees of freedom. Multiple R-squared: 0.0431, Adjusted R-squared: 0.003885. F-statistic: 1.099 on 5 and 122 DF, *p*-value: 0.3645. Of note, *p*-values of regression analyses are not Holm-corrected.

**Table S4.2**

**Regression model on syllables as reported for the right ear during the verbal dichotic listening task across both sessions.**

| **Coefficients** | **Estimate** | **Std. Error** | **t value** | **Pr(>\|t\|)** |
| --- | --- | --- | --- | --- |
| (Intercept) | 35.74 | 1.15 | 31.143 | < 0.001*** |
| Positive affect (PANAS) | -0.01 | 0.03 | -0.184 | 0.8545 |
| Negative affect (PANAS) | 0.00 | 0.06 | 0.046 | 0.9632 |
| Self-reported stressfulness (VAS) | -0.00 | 0.00 | -0.840 | 0.4024 |
| Cortisol | 0.02 | 0.01 | 1.917 | 0.0576 |
| sAA | -0.00 | 0.00 | -1.127 | 0.2618 |

*Note.* Table shows statistical parameters for a linear multiple regression model with syllables as reported for the right ear during the verbal dichotic listening task as outcome and stress and affect markers as predictors. Regression model was run across both sessions (online stressor and online control task). Stress and affect markers were included in form of AUCis. Signif. codes: 0 '***' 0.001 '**' 0.01 '*' 0.05 '.' 0.1 ' ' 1. Residual standard error: 8.259 on 122 degrees of freedom. Multiple R-squared: 0.04129, Adjusted R-squared: 0.001999. F-statistic: 1.051 on 5 and 122 DF, *p*-value: 0.391. Of note, *p*-values of regression analyses are not Holm-corrected.

**Table S4.3**

**Regression model on the lateralization quotient during the verbal dichotic listening task across both sessions.**

| **Coefficients** | **Estimate** | **Std. Error** | **t value** | **Pr(>\|t\|)** |
| --- | --- | --- | --- | --- |
| (Intercept) | 39.10 | 3.74 | 10.452 | < 0.001*** |
| Positive affect (PANAS) | 0.04 | 0.11 | 0.382 | 0.7031 |
| Negative affect (PANAS) | 0.11 | 0.18 | 0.609 | 0.5438 |
| Self-reported stressfulness (VAS) | -0.00 | 0.00 | -1.326 | 0.1875 |
| Cortisol | 0.06 | 0.03 | 2.059 | 0.0417* |
| sAA | -0.00 | 0.00 | -0.688 | 0.4931 |

*Note.* Table shows statistical parameters for a linear multiple regression model with the lateralization quotient as calculated for the verbal dichotic listening task as outcome and stress and affect markers as predictors. Regression model was run across both sessions (online stressor and online control task). Stress and affect markers were included in form of AUCis. Signif. codes: 0 '***' 0.001 '**' 0.01 '*' 0.05 '.' 0.1 ' ' 1. Residual standard error: 26.67 on 118 degrees of freedom. Multiple R-squared: 0.05042, Adjusted R-squared: 0.01018. F-statistic: 1.253 on 5 and 118 DF, *p*-value: 0.2889. Of note, *p*-values of regression analyses are not Holm-corrected.

**Table S4.4**

**Regression model on syllables as reported for the left ear during the verbal dichotic listening task for the online stressor only.**

| **Coefficients** | **Estimate** | **Std. Error** | **t value** | **Pr(>\|t\|)** |
| --- | --- | --- | --- | --- |
| (Intercept) | 15.00 | 1.85 | 8.107 | < 0.001*** |
| Positive affect (PANAS) | -0.06 | 0.03 | -1.898 | 0.0626 |
| Negative affect (PANAS) | -0.05 | 0.05 | -0.977 | 0.3327 |
| Self-reported stressfulness (VAS) | 0.00 | 0.00 | 0.725 | 0.4714 |
| Cortisol | -0.01 | 0.01 | -1.382 | 0.1722 |
| sAA | 0.00 | 0.00 | 0.363 | 0.718 |

*Note.* Table shows statistical parameters for a linear multiple regression model with syllables as reported for the left ear during the verbal dichotic listening task as outcome and stress and affect markers as predictors. Regression model was run for the online stressor only. Stress and affect markers were included in form of AUCis. Signif. codes: 0 '***' 0.001 '**' 0.01 '*' 0.05 '.' 0.1 ' ' 1. Residual standard error: 6.735 on 58 degrees of freedom. Multiple R-squared: 0.1018, Adjusted R-squared: 0.02432. F-statistic: 1.314 on 5 and 58 DF, *p*-value: 0.2708. Of note, *p*-values of regression analyses are not Holm-corrected.

**Table S4.5**

**Regression model on syllables as reported for the right during the verbal dichotic listening task for the online stressor only.**

| **Coefficients** | **Estimate** | **Std. Error** | **t value** | **Pr(>\|t\|)** |
| --- | --- | --- | --- | --- |
| (Intercept) | 33.83 | 2.40 | 14.122 | < 0.001*** |
| Positive affect (PANAS) | 0.04 | 0.04 | 0.926 | 0.358 |
| Negative affect (PANAS) | 0.00 | 0.07 | 0.068 | 0.946 |
| Self-reported stressfulness (VAS) | 0.00 | 0.00 | 0.305 | 0.761 |
| Cortisol | 0.02 | 0.01 | 1.667 | 0.101 |
| sAA | -0.00 | 0.00 | -0.693 | 0.491 |

*Note.* Table shows statistical parameters for a linear multiple regression model with syllables as reported for the right ear during the verbal dichotic listening task as outcome and stress and affect markers as predictors. Regression model was run for the online stressor only. Stress and affect markers were included in form of AUCis. Signif. codes: 0 '***' 0.001 '**' 0.01 '*' 0.05 '.' 0.1 ' ' 1. Residual standard error: 8.722 on 58 degrees of freedom. Multiple R-squared: 0.05426, Adjusted R-squared: -0.02727. F-statistic: 0.6655 on 5 and 58 DF, *p*-value: 0.6511. Of note, *p*-values of regression analyses are not Holm-corrected.

**Table S4.6**

**Regression model on the lateralization quotient during the verbal dichotic listening task for the online stressor only.**

| **Coefficients** | **Estimate** | **Std. Error** | **t value** | **Pr(>\|t\|)** |
| --- | --- | --- | --- | --- |
| (Intercept) | 37.02 | 7.27 | 5.090 | < 0.001*** |
| Positive affect (PANAS) | 0.20 | 0.13 | 1.546 | 0.1278 |
| Negative affect (PANAS) | 0.13 | 0.20 | 0.660 | 0.5122 |
| Self-reported stressfulness (VAS) | -0.00 | 0.00 | -0.399 | 0.6914 |
| Cortisol | 0.07 | 0.04 | 1.722 | 0.0906 |
| sAA | -0.00 | 0.00 | -0.439 | 0.6621 |

*Note.* Table shows statistical parameters for a linear multiple regression model with the lateralization quotient as calculated for the verbal dichotic listening task as outcome and stress and affect markers as predictors. Regression model was run for the online stressor only. Stress and affect markers were included in form of AUCis. Signif. codes: 0 '***' 0.001 '**' 0.01 '*' 0.05 '.' 0.1 ' ' 1. Residual standard error: 26.48 on 56 degrees of freedom. Multiple R-squared: 0.0893, Adjusted R-squared: 0.007987. F-statistic: 1.098 on 5 and 56 DF, *p*-value: 0.3717. Of note, *p*-values of regression analyses are not Holm-corrected.

**Table S4.7**

**Regression model on emotions as reported for the left ear during the emotional dichotic listening task across both sessions.**

| **Coefficients** | **Estimate** | **Std. Error** | **t value** | **Pr(>\|t\|)** |
| --- | --- | --- | --- | --- |
| (Intercept) | 17.07 | 0.64 | 26.514 | < 0.001*** |
| Positive affect (PANAS) | 0.04 | 0.02 | 2.087 | 0.0391* |
| Negative affect (PANAS) | -0.00 | 0.03 | -0.150 | 0.8812 |
| Self-reported stressfulness (VAS) | 0.00 | 0.00 | 0.796 | 0.4276 |
| Cortisol | 0.01 | 0.01 | 1.243 | 0.2164 |
| sAA | 0.00 | 0.00 | 1.441 | 0.1521 |

*Note.* Table shows statistical parameters for a linear multiple regression model with emotions as reported for the left ear during the emotional dichotic listening task as outcome and stress and affect markers as predictors. Regression model was run for the online stressor only. Stress and affect markers were included in form of AUCis. Signif. codes: 0 '***' 0.001 '**' 0.01 '*' 0.05 '.' 0.1 ' ' 1. Residual standard error: 4.567 on 116 degrees of freedom. Multiple R-squared: 0.06764, Adjusted R-squared: 0.02746. F-statistic: 1.683 on 5 and 116 DF, *p*-value: 0.1441. Of note, *p*-values of regression analyses are not Holm-corrected.

**Table S4.8**

**Regression model on emotions as reported for the right ear during the emotional dichotic listening task across both sessions.**

| **Coefficients** | **Estimate** | **Std. Error** | **t value** | **Pr(>\|t\|)** |
| --- | --- | --- | --- | --- |
| (Intercept) | 13.06 | 0.60 | 21.891 | < 0.001*** |
| Positive affect (PANAS) | -0.00 | 0.02 | -0.138 | 0.890 |
| Negative affect (PANAS) | 0.04 | 0.03 | 1.278 | 0.204 |
| Self-reported stressfulness (VAS) | -0.00 | 0.00 | -1.656 | 0.100 |
| Cortisol | -0.00 | 0.01 | -0.189 | 0.850 |
| sAA | -0.00 | 0.00 | -1.273 | 0.206 |

*Note.* Table shows statistical parameters for a linear multiple regression model with emotions as reported for the right ear during the emotional dichotic listening task as outcome and stress and affect markers as predictors. Regression model was run for the online stressor only. Stress and affect markers were included in form of AUCis. Signif. codes: 0 '***' 0.001 '**' 0.01 '*' 0.05 '.' 0.1 ' ' 1. Residual standard error: 4.232 on 116 degrees of freedom. Multiple R-squared: 0.03743, Adjusted R-squared: -0.004057. F-statistic: 0.9022 on 5 and 116 DF, *p*-value: 0.4822. Of note, *p*-values of regression analyses are not Holm-corrected.

**Table S4.9**

**Regression model on the** **lateralization quotient during the emotional dichotic listening task across both sessions.**

| **Coefficients** | **Estimate** | **Std. Error** | **t value** | **Pr(>\|t\|)** |
| --- | --- | --- | --- | --- |
| (Intercept) | -14.50 | 3.62 | -4.011 | < 0.001*** |
| Positive affect (PANAS) | -0.11 | 0.11 | -1.010 | 0.314668 |
| Negative affect (PANAS) | 0.18 | 0.17 | 1.045 | 0.297964 |
| Self-reported stressfulness (VAS) | -0.00 | 0.00 | -1.348 | 0.180273 |
| Cortisol | -0.03 | 0.03 | -1.053 | 0.294428 |
| sAA | -0.00 | 0.00 | -1.680 | 0.095639 |

*Note.* Table shows statistical parameters for a linear multiple regression model with the lateralization quotient as calculated for the emotional dichotic listening task as outcome and stress and affect markers as predictors. Regression model was run across both sessions (online stressor and online control task). Stress and affect markers were included in form of AUCis. Signif. codes: 0 '***' 0.001 '**' 0.01 '*' 0.05 '.' 0.1 ' ' 1. Residual standard error: 25.78 on 118 degrees of freedom. Multiple R-squared: 0.05521, Adjusted R-squared: 0.01517. F-statistic: 1.379 on 5 and 118 DF, *p*-value: 0.2371. Of note, *p*-values of regression analyses are not Holm-corrected.

**Table S4.10**

**Regression model on emotions as reported for the left ear during the emotional dichotic listening task for the online stressor only.**

| **Coefficients** | **Estimate** | **Std. Error** | **t value** | **Pr(>\|t\|)** |
| --- | --- | --- | --- | --- |
| (Intercept) | 18.13 | 1.36 | 13.284 | < 0.001*** |
| Positive affect (PANAS) | 0.02 | 0.02 | 0.763 | 0.449 |
| Negative affect (PANAS) | -0.01 | 0.04 | -0.211 | 0.834 |
| Self-reported stressfulness (VAS) | 0.00 | 0.00 | 0.034 | 0.973 |
| Cortisol | 0.01 | 0.01 | 0.975 | 0.334 |
| sAA | -0.00 | 0.00 | -0.299 | 0.766 |

*Note.* Table shows statistical parameters for a linear multiple regression model with emotions as reported for the left ear during the emotional dichotic listening task as outcome and stress and affect markers as predictors. Regression model was run for the online stressor only. Stress and affect markers were included in form of AUCis. Signif. codes: 0 '***' 0.001 '**' 0.01 '*' 0.05 '.' 0.1 ' ' 1. Residual standard error: 4.955 on 55 degrees of freedom. Multiple R-squared: 0.03377, Adjusted R-squared: -0.05407. F-statistic: 0.3844 on 5 and 55 DF, *p*-value: 0.8574. Of note, *p*-values of regression analyses are not Holm-corrected.

**Table S4.11**

**Regression model on emotions as reported for the right ear during the emotional dichotic listening task for the online stressor only.**

| **Coefficients** | **Estimate** | **Std. Error** | **t value** | **Pr(>\|t\|)** |
| --- | --- | --- | --- | --- |
| (Intercept) | 12.28 | 1.18 | 10.373 | < 0.001*** |
| Positive affect (PANAS) | -0.00 | 0.02 | -0.170 | 0.866 |
| Negative affect (PANAS) | 0.05 | 0.03 | 1.425 | 0.160 |
| Self-reported stressfulness (VAS) | -0.00 | 0.00 | -0.972 | 0.335 |
| Cortisol | 0.00 | 0.01 | 0.039 | 0.969 |
| sAA | -0.00 | 0.00 | -0.771 | 0.444 |

*Note.* Table shows statistical parameters for a linear multiple regression model with emotions as reported for the right ear during the emotional dichotic listening task as outcome and stress and affect markers as predictors. Regression model was run for the online stressor only. Stress and affect markers were included in form of AUCis. Signif. codes: 0 '***' 0.001 '**' 0.01 '*' 0.05 '.' 0.1 ' ' 1. Residual standard error: 4.299 on 55 degrees of freedom. Multiple R-squared: 0.04649, Adjusted R-squared: -0.04019. F-statistic: 0.5364 on 5 and 55 DF, *p*-value: 0.7478. Of note, *p*-values of regression analyses are not Holm-corrected.

**Table S4.12**

**Regression model on the** **lateralization quotient during the emotional dichotic listening task for the online stressor only.**

| **Coefficients** | **Estimate** | **Std. Error** | **t value** | **Pr(>\|t\|)** |
| --- | --- | --- | --- | --- |
| (Intercept) | -19.84 | 7.75 | -2.560 | < 0.05* |
| Positive affect (PANAS) | -0.08 | 0.14 | -0.544 | 0.5885 |
| Negative affect (PANAS) | 0.26 | 0.21 | 1.187 | 0.2403 |
| Self-reported stressfulness (VAS) | -0.00 | 0.00 | -0.653 | 0.5164 |
| Cortisol | -0.04 | 0.04 | -0.920 | 0.3616 |
| sAA | -0.00 | 0.00 | -0.398 | 0.6920 |

*Note.* Table shows statistical parameters for a linear multiple regression model with the lateralization quotient as calculated for the emotional dichotic listening task as outcome and stress and affect markers as predictors. Regression model was run for the online stressor only. Stress and affect markers were included in form of AUCis. Signif. codes: 0 '***' 0.001 '**' 0.01 '*' 0.05 '.' 0.1 ' ' 1. Residual standard error: 28.2 on 56 degrees of freedom. Multiple R-squared: 0.05833, Adjusted R-squared: -0.02574. F-statistic: 0.6938 on 5 and 56 DF, *p*-value: 0.6303. Of note, *p*-values of regression analyses are not Holm-corrected.

**Table S4.13**

**Regression model on the pseudoneglect for the line bisection task as preformed with the left hand for lines shifted to the left across both sessions.**

| **Coefficients** | **Estimate** | **Std. Error** | **t value** | **Pr(>\|t\|)** |
| --- | --- | --- | --- | --- |
| (Intercept) | -3.83 | 0.42 | -9.024 | < 0.001*** |
| Positive affect (PANAS) | 0.00 | 0.01 | 0.120 | 0.9043 |
| Negative affect (PANAS) | -0.03 | 0.02 | -1.539 | 0.1260 |
| Self-reported stressfulness (VAS) | 0.00 | 0.00 | 1.895 | 0.0602 |
| Cortisol | -0.00 | 0.00 | -0.982 | 0.3276 |
| sAA | 0.00 | 0.00 | 1.662 | 0.0988 |

*Note.* Table shows statistical parameters for a linear multiple regression model with the pseudoneglect for the line bisection task as performed with the left hand on lines shifted to the left as outcome and stress and affect markers as predictors. Regression model was run across both sessions (online stressor and online control task). Stress and affect markers were included in form of AUCis. Signif. codes: 0 '***' 0.001 '**' 0.01 '*' 0.05 '.' 0.1 ' ' 1. Residual standard error: 3.156 on 138 degrees of freedom. Multiple R-squared: 0.04499, Adjusted R-squared: 0.01039. F-statistic: 1.3 on 5 and 138 DF, *p*-value: 0.2674. Of note, *p*-values of regression analyses are not Holm-corrected.

**Table S4.14**

**Regression model on the pseudoneglect for the line bisection task as preformed with the left hand for lines at a central position across both sessions.**

| **Coefficients** | **Estimate** | **Std. Error** | **t value** | **Pr(>\|t\|)** |
| --- | --- | --- | --- | --- |
| (Intercept) | -2.20 | 0.39 | -5.570 | < 0.001*** |
| Positive affect (PANAS) | -0.01 | 0.01 | -0.737 | 0.46231 |
| Negative affect (PANAS) | 0.06 | 0.18 | -3.151 | < 0.01** |
| Self-reported stressfulness (VAS) | 0.00 | 0.00 | 2.631 | < 0.01** |
| Cortisol | -0.00 | 0.00 | -0.642 | 0.52204 |
| sAA | 0.00 | 0.00 | 0.882 | 0.37940 |

*Note.* Table shows statistical parameters for a linear multiple regression model with the pseudoneglect for the line bisection task as performed with the left hand for lines at a central position as outcome and stress and affect markers as predictors. Regression model was run across both sessions (online stressor and online control task). Stress and affect markers were included in form of AUCis. Signif. codes: 0 '***' 0.001 '**' 0.01 '*' 0.05 '.' 0.1 ' ' 1. Residual standard error: 2.928 on 138 degrees of freedom. Multiple R-squared: 0.07552, Adjusted R-squared: 0.04202. F-statistic: 2.255 on 5 and 138 DF, *p*-value: 0.05233. Of note, *p*-values of regression analyses are not Holm-corrected.

**Table S4.15**

**Regression model on the pseudoneglect for the line bisection task as preformed with the left hand for lines shifted to the right across both sessions.**

| **Coefficients** | **Estimate** | **Std. Error** | **t value** | **Pr(>\|t\|)** |
| --- | --- | --- | --- | --- |
| (Intercept) | 0.99 | 0.50 | 2.005 | < 0.05* |
| Positive affect (PANAS) | -0.01 | 0.01 | -1.061 | 0.2904 |
| Negative affect (PANAS) | -0.03 | 0.02 | -1.315 | 0.1906 |
| Self-reported stressfulness (VAS) | 0.00 | 0.00 | 0.844 | 0.4000 |
| Cortisol | -0.00 | 0.00 | -0.535 | 0.5938 |
| sAA | 0.00 | 0.00 | 0.739 | 0.4609 |

*Note.* Table shows statistical parameters for a linear multiple regression model with the pseudoneglect for the line bisection task as performed with the left hand on lines shifted to the right as outcome and stress and affect markers as predictors. Regression model was run across both sessions (online stressor and online control task). Stress and affect markers were included in form of AUCis. Signif. codes: 0 '***' 0.001 '**' 0.01 '*' 0.05 '.' 0.1 ' ' 1. Residual standard error: 3.681 on 138 degrees of freedom. Multiple R-squared: 0.0212, Adjusted R-squared: -0.01426. F-statistic: 0.5979 on 5 and 138 DF, *p*-value: 0.7016. Of note, *p*-values of regression analyses are not Holm-corrected.

**Table S4.16**

**Regression model on the pseudoneglect for the line bisection task as preformed with the right hand for lines shifted to the left across both sessions.**

| **Coefficients** | **Estimate** | **Std. Error** | **t value** | **Pr(>\|t\|)** |
| --- | --- | --- | --- | --- |
| (Intercept) | -2.59 | 0.42 | -6.23 | < 0.001*** |
| Positive affect (PANAS) | -0.03 | 0.01 | -2.275 | 0.0244* |
| Negative affect (PANAS) | -0.02 | 0.02 | -0.984 | 0.3269 |
| Self-reported stressfulness (VAS) | -0.00 | 0.00 | -1.401 | 0.1636 |
| Cortisol | 0.00 | 0.00 | 0.646 | 0.5192 |
| sAA | 0.00 | 0.00 | 0.040 | 0.9678 |

*Note.* Table shows statistical parameters for a linear multiple regression model with the pseudoneglect for the line bisection task as performed with the right hand on lines shifted to the left as outcome and stress and affect markers as predictors. Regression model was run across both sessions (online stressor and online control task). Stress and affect markers were included in form of AUCis. Signif. codes: 0 '***' 0.001 '**' 0.01 '*' 0.05 '.' 0.1 ' ' 1. Residual standard error: 3.086 on 138 degrees of freedom. Multiple R-squared: 0.06071, Adjusted R-squared: 0.02668. F-statistic: 1.784 on 5 and 138 DF, *p*-value: 0.1201. Of note, *p*-values of regression analyses are not Holm-corrected.

**Table S4.17**

**Regression model on the pseudoneglect for the line bisection task as preformed with the right hand for lines at a central position across both sessions.**

| **Coefficients** | **Estimate** | **Std. Error** | **t value** | **Pr(>\|t\|)** |
| --- | --- | --- | --- | --- |
| (Intercept) | -1.22 | 0.49 | -2.503 | < 0.05* |
| Positive affect (PANAS) | -0.02 | 0.01 | -1.218 | 0.2253 |
| Negative affect (PANAS) | -0.00 | 0.02 | -0.076 | 0.9393 |
| Self-reported stressfulness (VAS) | -0.00 | 0.00 | -0.561 | 0.5760 |
| Cortisol | -0.00 | 0.00 | -0.999 | 0.3197 |
| sAA | 0.00 | 0.00 | 0.588 | 0.5573 |

*Note.* Table shows statistical parameters for a linear multiple regression model with the pseudoneglect for the line bisection task as performed with the right hand on at a central position as outcome and stress and affect markers as predictors. Regression model was run across both sessions (online stressor and online control task). Stress and affect markers were included in form of AUCis. Signif. codes: 0 '***' 0.001 '**' 0.01 '*' 0.05 '.' 0.1 ' ' 1. Residual standard error: 3.625 on 138 degrees of freedom. Multiple R-squared: 0.01954, Adjusted R-squared: -0.01598. F-statistic: 0.5501 on 5 and 138 DF, *p*-value: 0.738. Of note, *p*-values of regression analyses are not Holm-corrected.

**Table S4.18**

**Regression model on the pseudoneglect for the line bisection task as preformed with the right hand for lines shifted to the right across both sessions.**

| **Coefficients** | **Estimate** | **Std. Error** | **t value** | **Pr(>\|t\|)** |
| --- | --- | --- | --- | --- |
| (Intercept) | 2.28 | 0.62 | 3.693 | < 0.001*** |
| Positive affect (PANAS) | -0.01 | 0.02 | -0.699 | 0.485449 |
| Negative affect (PANAS) | -0.01 | 0.03 | -0.388 | 0.698356 |
| Self-reported stressfulness (VAS) | 0.00 | 0.00 | 0.489 | 0.625291 |
| Cortisol | 0.00 | 0.01 | 0.658 | 0.511359 |
| sAA | 0.00 | 0.00 | 0.824 | 0.411326 |

*Note.* Table shows statistical parameters for a linear multiple regression model with the pseudoneglect for the line bisection task as performed with the right hand on lines shifted to the right as outcome and stress and affect markers as predictors. Regression model was run across both sessions (online stressor and online control task). Stress and affect markers were included in form of AUCis. Signif. codes: 0 '***' 0.001 '**' 0.01 '*' 0.05 '.' 0.1 ' ' 1. Residual standard error: 4.589 on 138 degrees of freedom. Multiple R-squared: 0.01956, Adjusted R-squared: -0.01596. F-statistic: 0.5507 on 5 and 138 DF, *p*-value: 0.7375. Of note, *p*-values of regression analyses are not Holm-corrected.

**Table S4.19**

**Regression model on the pseudoneglect for the line bisection task as preformed with the left hand for lines shifted to the left for the online stressor only.**

| **Coefficients** | **Estimate** | **Std. Error** | **t value** | **Pr(>\|t\|)** |
| --- | --- | --- | --- | --- |
| (Intercept) | -4.17 | 0.92 | -4.551 | < 0.001*** |
| Positive affect (PANAS) | 0.01 | 0.01 | 0.583 | 0.5617 |
| Negative affect (PANAS) | -0.03 | 0.02 | -1.330 | 0.1880 |
| Self-reported stressfulness (VAS) | 0.00 | 0.00 | 1.299 | 0.1984 |
| Cortisol | -0.00 | 0.00 | -0.954 | 0.3434 |
| sAA | 0.00 | 0.00 | 2.137 | < 0.05* |

*Note.* Table shows statistical parameters for a linear multiple regression model with the pseudoneglect for the line bisection task as performed with the left hand on lines shifted to the left as outcome and stress and affect markers as predictors. Regression model was run for the online stressor only. Stress and affect markers were included in form of AUCis. Signif. codes: 0 '***' 0.001 '**' 0.01 '*' 0.05 '.' 0.1 ' ' 1. Residual standard error: 3.307 on 66 degrees of freedom. Multiple R-squared: 0.09258, Adjusted R-squared: 0.02383. F-statistic: 1.347 on 5 and 66 DF, *p*-value: 0.2559. Of note, *p*-values of regression analyses are not Holm-corrected.

**Table S4.20**

**Regression model on the pseudoneglect for the line bisection task as preformed with the left hand for lines at a central position for the online stressor only.**

| **Coefficients** | **Estimate** | **Std. Error** | **t value** | **Pr(>\|t\|)** |
| --- | --- | --- | --- | --- |
| (Intercept) | -2.62 | 0.88 | -2.966 | < 0.01** |
| Positive affect (PANAS) | -0.01 | 0.01 | -0.978 | 0.3315 |
| Negative affect (PANAS) | -0.05 | 0.02 | -2.399 | < 0.05* |
| Self-reported stressfulness (VAS) | 0.00 | 0.00 | 1.677 | 0.0983 |
| Cortisol | -0.00 | 0.00 | -0.251 | 0.8027 |
| sAA | 0.00 | 0.00 | 0.859 | 0.3932 |

*Note.* Table shows statistical parameters for a linear multiple regression model with the pseudoneglect for the line bisection task as performed with the left hand for lines at a central position as outcome and stress and affect markers as predictors. Regression model was run for the online stressor only. Stress and affect markers were included in form of AUCis. Signif. codes: 0 '***' 0.001 '**' 0.01 '*' 0.05 '.' 0.1 ' ' 1. Residual standard error: 3.187 on 66 degrees of freedom. Multiple R-squared: 0.08842, Adjusted R-squared: 0.01936. F-statistic: 1.28 on 5 and 66 DF, *p*-value: 0.283. Of note, *p*-values of regression analyses are not Holm-corrected.

**Table S4.21**

**Regression model on the pseudoneglect for the line bisection task as preformed with the left hand for lines shifted to the right for the online stressor only.**

| **Coefficients** | **Estimate** | **Std. Error** | **t value** | **Pr(>\|t\|)** |
| --- | --- | --- | --- | --- |
| (Intercept) | 1.41 | 1.04 | 1.356 | 0.1796 |
| Positive affect (PANAS) | -0.03 | 0.02 | -1.892 | 0.0629 |
| Negative affect (PANAS) | -0.02 | 0.03 | -0.913 | 0.3643 |
| Self-reported stressfulness (VAS) | 0.00 | 0.00 | 0.033 | 0.9735 |
| Cortisol | -0.00 | 0.01 | -0.461 | 0.6460 |
| sAA | 0.00 | 0.00 | 0.234 | 0.8159 |

*Note.* Table shows statistical parameters for a linear multiple regression model with the pseudoneglect for the line bisection task as performed with the left hand on lines shifted to the right as outcome and stress and affect markers as predictors. Regression model was run for the online stressor only. Stress and affect markers were included in form of AUCis. Signif. codes: 0 '***' 0.001 '**' 0.01 '*' 0.05 '.' 0.1 ' ' 1. Residual standard error: 3.738 on 66 degrees of freedom. Multiple R-squared: 0.05523, Adjusted R-squared: -0.01634. F-statistic: 0.7717 on 5 and 66 DF, *p*-value: 0.5735. Of note, *p*-values of regression analyses are not Holm-corrected.

**Table S4.22**

**Regression model on the pseudoneglect for the line bisection task as preformed with the right hand for lines shifted to the left for the online stressor only.**

| **Coefficients** | **Estimate** | **Std. Error** | **t value** | **Pr(>\|t\|)** |
| --- | --- | --- | --- | --- |
| (Intercept) | -1.60 | 0.80 | -1.989 | 0.0508 |
| Positive affect (PANAS) | -0.03 | 0.01 | -2.040 | < 0.05* |
| Negative affect (PANAS) | -0.01 | 0.02 | -0.518 | 0.6062 |
| Self-reported stressfulness (VAS) | -0.00 | 0.00 | -2.200 | < 0.05* |
| Cortisol | -0.00 | 0.00 | -0.215 | 0.8306 |
| sAA | 0.00 | 0.00 | 0.433 | 0.6667 |

*Note.* Table shows statistical parameters for a linear multiple regression model with the pseudoneglect for the line bisection task as performed with the right hand on lines shifted to the left as outcome and stress and affect markers as predictors. Regression model was run for the online stressor only. Stress and affect markers were included in form of AUCis. Signif. codes: 0 '***' 0.001 '**' 0.01 '*' 0.05 '.' 0.1 ' ' 1. Residual standard error: 2.902 on 66 degrees of freedom. Multiple R-squared: 0.1438, Adjusted R-squared: 0.07889. F-statistic: 2.216 on 5 and 66 DF, *p*-value: 0.06293. Of note, *p*-values of regression analyses are not Holm-corrected.

**Table S4.23**

**Regression model on the pseudoneglect for the line bisection task as preformed with the right hand for lines at a central position for the online stressor only.**

| **Coefficients** | **Estimate** | **Std. Error** | **t value** | **Pr(>\|t\|)** |
| --- | --- | --- | --- | --- |
| (Intercept) | -0.03 | 0.99 | -0.031 | 0.976 |
| Positive affect (PANAS) | -0.02 | 0.02 | -1.385 | 0.171 |
| Negative affect (PANAS) | -0.00 | 0.03 | -0.066 | 0.948 |
| Self-reported stressfulness (VAS) | -0.00 | 0.00 | -1.085 | 0.282 |
| Cortisol | -0.01 | 0.01 | -1.017 | 0.313 |
| sAA | -0.00 | 0.00 | -0.369 | 0.713 |

*Note.* Table shows statistical parameters for a linear multiple regression model with the pseudoneglect for the line bisection task as performed with the right hand on at a central position as outcome and stress and affect markers as predictors. Regression model was run for the online stressor only. Stress and affect markers were included in form of AUCis. Signif. codes: 0 '***' 0.001 '**' 0.01 '*' 0.05 '.' 0.1 ' ' 1. Residual standard error: 3.559 on 66 degrees of freedom. Multiple R-squared: 0.05045, Adjusted R-squared: -0.02148. F-statistic: 0.7014 on 5 and 66 DF, *p*-value: 0.6244. Of note, *p*-values of regression analyses are not Holm-corrected.

**Table S4.24**

**Regression model on the pseudoneglect for the line bisection task as preformed with the right hand for lines shifted to the right for the online stressor only.**

| **Coefficients** | **Estimate** | **Std. Error** | **t value** | **Pr(>\|t\|)** |
| --- | --- | --- | --- | --- |
| (Intercept) | 3.12 | 1.28 | 2.428 | < 0.05* |
| Positive affect (PANAS) | -0.02 | 0.02 | -1.004 | 0.3192 |
| Negative affect (PANAS) | -0.01 | 0.03 | -0.173 | 0.8628 |
| Self-reported stressfulness (VAS) | 0.00 | 0.00 | 0.030 | 0.9759 |
| Cortisol | 0.00 | 0.00 | 0.592 | 0.5555 |
| sAA | -0.00 | 0.00 | -0.863 | 0.3915 |

*Note.* Table shows statistical parameters for a linear multiple regression model with the pseudoneglect for the line bisection task as performed with the right hand on lines shifted to the right as outcome and stress and affect markers as predictors. Regression model was run for the online stressor only. Stress and affect markers were included in form of AUCis. Signif. codes: 0 '***' 0.001 '**' 0.01 '*' 0.05 '.' 0.1 ' ' 1. Residual standard error: 4.634 on 66 degrees of freedom. Multiple R-squared: 0.03436, Adjusted R-squared: -0.03879. F-statistic: 0.4697 on 5 and 66 DF, *p*-value: 0.7975. Of note, *p*-values of regression analyses are not Holm-corrected.
